# Joint SASMA and Bricscess Congress: Cape Town - South Africa, Thursday 10 – Sunday 13 October 2019

**Published:** 2019-01-01

Despite all efforts, the health of today's youth continues to decline. The problem is that two classes, or even one class, per week with a physical education teacher is not enough to form a habit and obviously there is not enough time for both developing motor skills and improving the health conditions of students. Some of the students require more time and more effort from physical education teacher during the classes. certain conditions of the students health must be taking into account during the physical education process, for example cardiovascular disease. Based on the collected data our physical education teachers and IT department of the University build an online platform when a student can have online PE classes at home. The student must login to the web page of the academy then he can choose from a number of lectures according to his physical conditions. He can start to do exercises according to the video guidance of the lesson. The website contains all necessary theoretical information for students to be able to form motivation and build healthy habits, be physically active on their own. The teacher can see how much time did the student spend on the website as well he can give to the student multiple choice question tests to be sure that the theoretical information assimilated by the student. The results of the experiment were provided to be statistically different shows us that just physical education classes as well as just independent online classes are not working separately we need to have a joined efforts the physical education teachers and the pupils to get the results. Physical education proses must include activities outside the classroom and internet technologies give us opportunities to make the education process more useful and meaningful for promoting the healthy lifestyle among young people, as a result, it will improve the quality of their life and make their life longer and better.

