# Supplementary material for: Joint SASMA and Bricscess Congress: Cape Town - South Africa, Thursday 10 – Sunday 13 October 2019
Source: S Afr J Sports Med. 2019 Jan 1;31(1):v31i1a7472. (PMC9924542)
Supplement: Related texts

# Abstract: I02: Prediction of Human Performance in Sports

**Published:** 2019-01-01

**Authors:** G. Balasekaran

**Affiliations:** Physical Education & Sports Science, National Institute of Education, Nanyang Technological University, Singapore

Various sports require a combination of anaerobic and aerobic energy, thus the measurement of anaerobic peak energy or anaerobic performance is an important parameter of sports activities. The measurement of aerobic energy is well-researched and there is a standardised method; however, there are too many different methods to measure anaerobic performances such as blood lactate, maximal accumulated oxygen deficit (MAOD), oxygen debt, muscle biopsy etc. Moreover, it is essential to utilise a method that is reliable, valid, easy and convenient. MAOD may be an accurate method but it is not easy and convenient to measure anaerobic performance. Besides physiological methods, mathematical models may be used to measure anaerobic energy. As individual athletes come from different physiological backgrounds, they can be categorized according to their capacities in endurance and sprint capabilities. Bundle et.al.(2003) developed the concept of Anaerobic Speed Reserve (AnSR) to measure anaerobic performance accurately. However, AnSR can only be used to predict 3 to 240 seconds. Therefore, a more accurate model incorporating timings greater than 240 seconds may be needed. A recently developed mathematical model called running energy reserve index (RERI) may be a solution. RERI has been able to significantly differentiate athletes of different training backgrounds. Therefore, RERI may be an accurate and non-invasive method to categorize athletes from different physiological backgrounds.

# Abstract: I03: The Linkage of Holistic Health to UNSDG 17: Spanish Perspective

**Published:** 2019-01-01

**Authors:** Myriam GUERRA-BALIC, Verónica Violant HOLZ

**Affiliations:** 1 Principal Investigator of the Research Group on Health, Physical Activity and Sport, FPCEE-Blanquerna, University Ramon Llull, Spain; 2 Director of Department of Didactic and Educational Organization, University of Barcelona Spain

Usually, when we think about the concept of disease, we relate it to a loss of health. But talking about health we should consider its determinants at the same time that the factors which can condition at the time that can facilitate both the individual and collective health state. UNESCO (2015) advices us about the importance to obtain 17 sustainable Development Goals included in the Agenda 2030, on five of which we will focus: Goal 3. Good Health and Well-Being; Goal 4. Quality Education; Goal 10. Reduced Inequalities; Goal 11. Sustainable Cities and Communities; and Goal 17. Partnership for the Goals. Planning these actions to achieve the goals from a health point of view implies designing good practices addressed to children and adolescents, and their families, in order to manage and transform the risk factors into options to promote healthy lifestyle. With all this, we can afford several collectives with risk due to different factors that can be related to the previous cited goals. Our objective is to analyze the elements of these goals that pass on good practices, and to propose future actions that can be visualized in several environments (school, hospitals, homes, social places, community, etc.) and that guarantee the sustainability and support networks like the Foundation For Global Community Health (GCH). This foundation has the following purposes: “to develop best practices regarding school based well-being programs, utilizing the whole school, whole community and whole child in cooperation with an international network of advisers” (GCH, 2017).

# Abstract: I04: Health Policy Approach to Address Injuries among Children Participating in Sports

**Published:** 2019-01-01

**Authors:** Jingzhen (Ginger) Yang

**Affiliations:** Pediatrics and Epidemiology, Nationwide Children’s Hospital, The Ohio State University, USA/China

Sports and recreational activities are widely promoted as part of a healthy lifestyle for children. However, the high incidence of injuries among children participating in these activities could diminish the physical and psychological benefits gained from participating and pose a serious threat to the health and well-being of children. Each year, at least 4.3 million sports and recreational injuries occur to school-aged children in the United States (US), with approximately 2 million being sports-related concussions, a form of traumatic brain injury (TBI). Concussions are of particular concern for children since the brain is still developing during this critical period. Untreated or improperly managed concussions can have longlasting, detrimental effects on young brains, affecting the child’s physical, cognitive, emotional, and sleep health. Numerous efforts have been made in the US to prevent sports and recreational injuries, including sports-related concussions. One such effort to mitigate negative health consequences is a public health policy approach. In this presentation, an approach to address youth TBI prevention and control will be introduced and discussed. Examples on state-level youth TBI laws will be used to illustrate how a public health policy approach is used to address sports-related concussion prevention and control. The presentation will cover what state-level youth TBI laws are, how these laws are implemented and enforced at local schools, how these laws are evaluated using data from a large, national sports-injury surveillance system, and what the impact of these laws is on the trends of new and recurrent concussion rates among US representative high-school athletes while participating in the 9 most commonly played sports across a period of 11 years (2006–2016) from pre- to post-law enactment. These results, along with those of others, suggest that more public health policy efforts are needed to focus on preventing concussions in the first place, such as preventing or reducing initial head or body impact among children who participate in sports and recreational activities.

# Abstract: I05: The Connection of Cognitive Function and Physical Activity in Students: Current Research and Implication

**Published:** 2019-01-01

**Authors:** Gıyasettin DEMIRHAN

**Affiliations:** Head of Physical Education and Sport Education Department, Faculty of Sports Sciences, Hacettepe University, Turkey

The purpose of this paper is to explain the connection of cognitive function (CF) and physical activity (PA) in children and young people. It will be shared some research results about mental functions with this paper. A growing number of studies support the idea that physical exercise is a lifestyle factor that might lead to increased physical and mental health throughout life (Hillman et. al., 2008). As we know that physical activity refers to all forms activity that requires physical effort, from simple house tasks to performance sports. This paper focuses on fundamental and specific movement skills, exercises and sport skills about the students at schools from elementary school to the university. Research has suggested that physical activity might not only support to improve their physical health, but might also develop their cognitive functions. According to many research results, physical activity has important roles on improving mental functions, raising academic performance and developing positive attitude. For instance, physical activity promotes children’s and young people’s health and well-being in many ways. It has also been noted to have a positive effect on learning and cognitive functions, such as memory and executive functions and, as a result, possibly on academic performance (Hilmann et.al., 2009; Kwak et. al., 2009; Tomprowski et. al., 2008). Furthermore, many previously conducted studies have demonstrated that all forms of physical activity have a positive effect on the academic performance of children, and on the development of their cognitive characteristics. Physical activity also affects development of positive attitude. (Koca and Demirhan, 2004; Hunuk and Demirhan, 2010; Cairney et al., 2012).

# Abstract: I06: An analysis of the implementation of the national sport and recreation plan in the Western Cape

**Published:** 2019-01-01

**Authors:** Lyndon Bouah

**Affiliations:** Chief Director for Sport and Recreation, Department of Cultural Affairs and Sport, South Africa

It is widely accepted that sport has the potential to serve as a tool for development as well as a catalyst for social and personal change. The National Department of Sport and Recreation (SRSA) has recognized the role that sport can play in building a better society in its recently published document the National Sport and Recreation Plan (NSRP). The study analysed the implementation of the NSRP in the Western Cape and developed options for the improvement of the implementation of the National Sport and Recreation Plan in the Western Cape. The study analysed the factors that are necessary for an enabling environment to be successful to create an active and winning nation. By linking policy to implementation the study showed that the social benefits of sport can be extended to the broader social community. The fieldwork research was conducted with sport councils, sport federations, academy officials and senior government officials. The study found that the links between local, provincial and national government must be strengthened. The study further found that the level of commitment amongst the various spheres of government is not the same. The will may be there but the actual resources that must be committed is not readily made available. The 5-C protocol was used to analyse the NSRP and its current implementation within the Western Cape. Communication was considered as a future addition to augment the 5-C protocol. The alignment between various national, provincial and local spheres of government was found to be lacking. It is recommended that the NSRP be brought into the annual performance plans, strategic plans, medium term expenditure framework and performance agreements of departments and senior management. It was found that the voluntary nature of organisations may act as a hindrance to the implementation of the NSRP. It is foreseen that the outcome of the study will assist in benchmarking best practices for implementation. The recommendations proposed by this study will provide options for laying the foundation for the successful implementation of the National Sport and Recreation Plan in South Africa.

# Abstract: I07: Physical Activity, Exercise, Blood Lipids and Lipoproteins: Implications for Prevention of Chronic Metabolic Disorders

**Published:** 2019-01-01

**Authors:** Larry DURSTINE

**Affiliations:** Distinguished Professor, Department of Exercise Science, University of South Carolina, USA

The beneficial effects of regular exercise participation on plasma lipid and lipoprotein profiles have in recent years have been more clearly defined. Presently, the many factors that influence lipid synthesis and catabolism, the interactions between lipids, lipoproteins, apolipoproteins (apo), and lipoprotein enzymes are better understood. Though much has been learned regarding the plasma lipids, the various components of the different lipoproteins, and the various genetic and environmental factors that impact the lipoprotein metabolic pathways and eventually lipoprotein composition, many new questions remain unanswered. Gender, aging, body fat distribution, dietary composition, cigarette smoking status, and regular exercise participation are factors that interact to modify lipoprotein metabolism and plasma lipoprotein composition. For example, intervention programs that reduce dietary fat and increase dietary carbohydrate while increasing daily physical activity can positively influence plasma lipid and lipoprotein concentrations and reduce coronary artery disease risk. This presentation will summarize the beneficial impact of exercise on blood lipid and lipoproteins.

# Abstract: I08: Practical approach of sport injuries prevention – a Romanian perspective

**Published:** 2019-01-01

**Authors:** Luminita GEORGESCU

**Affiliations:** Department of Physical Education and Sport, Faculty of Sciences, Physical Education and Informatics, University of Pitesti, Romania

## Introduction

With the continuous increase in the number of practitioners, currently there is also a significant increase in the risk of injury due to sport activities, whether they is for performance or leisure. Continuous adaptations and improvements of the equipment, of the training techniques and regulations are some of the concerns that specialists in the field of sport do have in respect with the prevention of specific injuries.

## Methods

Health status screening, medical forms and certificates to approve sports participation are some of the methods used by the sports medicine staff to prevent injuries and the risk of injury. We used a retrospective longitudinal study and analyzed the medical records of 1200 athletes (300 playing individual sports and 900 practicing sports games) over a period of five years. Through a structured interview we gathered data of the athletes’ health status and also their injury records during training and competition. We were interested also to range the athletes’ characteristics depending on their anthropometric measurements and physical development, clinical and paraclinical examinations and aerobic and anaerobic exercise capacity. Our aim was to identify the biological profile of athletes at high risk of injury and the correlation of individual sanogenic factors with the sport’s requirements, respectively the training stage. Starting from this, we wanted to project an intervention strategy, based on a combination of methods to increase the fitness level, pharmacological treatment, hygiene-dietetic measures and physiotherapy programs.

## Results & Discussion

After processing all data, some injury-associated risk factors were identified: deficiencies in the athletes’ selection by sports branches; inconsistency between the physical development of the athletes and the sport requirements; paucity of the body’s ability to adapt to the physical effort according to the stage of sports training. Conclusion. There is a close correlation between the initial clinical and paraclinical assessment and then the periodical assessment in determining the athlete's health status and his effort adaptation ability. Moreover, the correction of biochemical imbalances has a special role in preventing the risk of injuries.

# Abstract: I09: Is teaching the use of BMI to classify obesity and overweight outdated?

**Published:** 2019-01-01

**Authors:** Hans De Ridder

**Affiliations:** Professor and Director, School of Human Movement Sciences, North-West University, Potchefstroom, South Africa

## Introduction

Among many weight-to-height ratios, the body mass index may be the most common index. While body mass index was proposed in 1972, the equation-mass (kg)/stature (m)_2 - was originally proposed in 1832 and named the Quetelet Index. In 1997, the World Health Organization chose to utilize body mass index as an index to assess overweight and obesity in adults and the international classification scheme was proposed. Although body mass index was not originally proposed to identify obesity, body mass index has a quadratic relationship with percentage body fat in both genders.

## Methods

Several large-scale epidemiological studies reported that body mass index is positively associated with a number of obesity-related chronic diseases, including cardiovascular diseases and type ll diabetes mellitus. While body mass index has been utilized in assessments of overweight and obesity, it is important to appreciate that it is simply a measure of heaviness, not of fatness, as it does not use any measures of body composition. A classic study by Garn suggested three limitations of body mass index: (1) body mass index is stature dependent and in different directions at different points in the life cycle; (2) body mass index may be affected by relative leg length or relative sitting height; and (3) body mass index may reflect both lean and fat tissues to a comparable degree.

## Conclusion

Many studies have reported misclassification of individuals and variability in the relationship between body mass index and percentage body fat due to these limitations. Such limitations are applicable to other weight-to-height ratio and therefore it is important to use these indices with caution, particularly when the index was applied to individuals and special population. For a better use of body mass index to different racial groups the World Health Organization expert consultation proposed new cut-off points (i.e. 23 kg/m_2, 27.5 kg/m_2, 32.5 kg/m_2 and 37.5 kg/m_2) as public health action points in 2004.

# Abstract: I10: Creating a Sporting Culture in India for Health Promotion and Achieving Excellence in Sports

**Published:** 2019-01-01

**Authors:** Gulshan KHANNA

**Affiliations:** Director Project Indian Institute of Sports Science and Research Ministry of Youth Affairs and Sports, Dean, Faculty of Applied Sciences, Manav Rachna International University, India

India has so far won a total of 28 medals – with 88% of its gold medals coming from hockey alone since 1900. Despite its immense talent base and growing economic prowess in recent years, historical legacies rooted in our byzantine sporting system and a general absence of a physical activity culture have meant that India has so far been unable to produce elite-level global success in sports that is commensurate to its huge potential, size and role as a rising power. On the initiative of Prime Minister Olympics Task Force was appointed by the union Ministry of Youth Affairs and Sports to recommend systemic steps for improved Indian performances at the 2020, 2024 and 2028 Olympics and to create a road map for realizing the huge potential of Indian sports and improve health through sports. A detailed feedback from all stakeholders in Indian sport, through various meetings held in state capitals, met several athletes and para-athletes and studied health promotion, physical activity, sporting culture undertaken in other countries for international benchmarking to understand global best practices and whether they can be adapted to reform the Indian system of health promotion and sports improvement. Various steps have been recommended to improve the physical activity and create a sporting culture for improving health promotion in India to achieve excellence in Sports.

# Abstract: I11: The Sustainability of High-Quality Physical Education Programming

**Published:** 2019-01-01

**Authors:** Kim GRABER, Gabriella McLoughlin

**Affiliations:** 1 Associate Head, Department of Kinesiology and Community Health, Director, Campus Honors Program, The University of Illinois at Urbana–Champaign, USA

Approximately 15% of children (aged 2–19 years) in the United States are classed as overweight, with an additional 17% classed as obese (Ogden, Carroll, Kit, & Flegal, 2012). In order to address the obesity epidemic, K-12 schools in the United States have been encouraged to develop and implement a Comprehensive School Physical Activity Program (CSPAP) that can be infused into the school curriculum in order to increase opportunities for children to be physically active. This multi-dimensional model includes quality physical education, which serves as the cornerstone, along with before and after school physical activity opportunities, physical activity within the school, staff involvement in the program, and community engagement. Physical educators have been encouraged to take an active role in facilitating the CSPAP initiative, and professional organizations support its implementation in schools throughout the US. Unfortunately, there is a paucity of research regarding the effectiveness of CSPAPs, and only one study addresses the effectiveness of those programs that have been deemed successful or have received national recognition for their efforts (McLoughlin & Graber, in review). In the one study that has been conducted, it is clear that sustaining the quality of such programs is difficult, particularly as a result of administrative and staff turnover and little time for physical education colleagues to communicate with each other about program maintenance. Although the successful development of an individual school CSPAP is often the result of the efforts of only one person, or a small group of individuals, it requires continuous support from multiple stakeholders within and outside the school community to sustain the quality of the program. Grounded in the social ecological model (Bronfenbrenner, 1989; Stokols, 1992), this presentation will address the difficulty of sustainability of high quality physical education, particularly within an educational microcosm that for many years has been accepting of low quality physical education across the nation.

# Abstract: I12: Dancing with the World: Aim for Globalize Harmony with Holistic Health through Performing and Education

**Published:** 2019-01-01

**Authors:** Miranda CHIN

**Affiliations:** Artistic Director, Miranda Chin Dance Company Principal, Danceland School, Hong Kong- China

The Chinese culture and philosophy got over 5,000 years in history, while Martial Arts and Tai Chi is one of the most representative elements in it. Through applying these elements into simplified contemporary dances, the general public can easily get in touch with dances and getting good health and be able to sense the spirit of Chinese culture particularly the feeling of harmony with the nature. Chin had produced eight series of martial art and dances for reflecting the source of the martial arts, principle of movement, Yi philosophy, the five elements of nature, health and living philosophy. Chin will explain the creation process of these eight series of dance including how she did the research and development. We see and feel then we dance, we dance to show what we see and feel, that means dance is a body language. It is therefore dances are the history of people's culture and philosophy. Thus Chin's dance tells the contemporary ways, shows the culture and spirit of Chinese. Through explanation, video, this presentation may lead students and professionals to understand what Chin's dances bring to them …… Chinese culture, Chinese philosophy, good health, spiritual state via contemporary dynamic movement. Then she will share her concept and process of her recent artistic development is on mergering Chinese culture into simplifed dances for promoting Chinese culture and health and is now has over 56 countries applying it.

# Abstract: I13: Reform in Physical Education and Sport in Brazil: New Perspectives and Possibilities of Best Practices

**Published:** 2019-01-01

**Authors:** Nara OLIVEIRA

**Affiliations:** Head Human Movement Sciences Department, Federal University of Sao Paulo, Brazil

Physical Education (PE) in Brazil was introduced under eugenic and hygienic principles, in the early 20th century, when became a mandatory in the school curriculum. On the first half of that century, gymnastics was the main content, derived from European methods. The Sport became relevant on the school curriculum after the 1960's, during the military regime, as a way to social control. The return of democracy in 1980's opens a new chapter to PE and Sport, with the curriculum reform. New PE and Sport paradigms were set up, under the educational and cultural perspective. In the 1990’s, curricular guidelines has published by Brazilian Ministry of Education, highlighting PE as relevant to the human development, as well the Sport, through the curricular or extra curricular activities, both inside or outside school. Over the last 20 years, public policies from Brazilian Ministry of Education, Ministry of Sports and also Ministry of Health has been developed, focusing PE and Sport as a tool for human education in a holistic perspective. Additionally, the investment on PE teacher training and research in the area has also been increased, to developing best pedagogical practices. However, the way forward is a great challenge, considering the cultural and economic Brazilian diversity. In this context, the purpose of this presentation is to provide data regarding the new perspectives and possibilities of best practices, in the face of the Brazilian PE and Sport reform over the last years.

# Abstract: I14: Do Antioxidant Supplements Improve Sports Performance and Health? : A Malaysian Perspective

**Published:** 2019-01-01

**Authors:** Chee Keong Chen

**Affiliations:** Exercise and Sports Science Programme, School of Health Sciences, Universiti Sains Malaysia, 16150 Kubang Kerian, Kelantan, Malaysia

Free radicals are reactive oxygen species which are highly reactive because of an unpaired valence electron. They are capable of damaging healthy cells and thus have been implicated in the pathophysiology of many diseases such as cancer, atherosclerosis, rheumatoid arthritis and neurodegenerative disease. Antioxidants, on the other hand, are substances that scavenge free radicals and offer protection from the damaging effects of free radicals. Nevertheless, oxidative stress occurs when the rate of production of free radicals exceeds the body’s antioxidant capacity to detoxify them. Research data demonstrating significant increases in F_2_ isoprostanes and lipid hydroperoxides following exhaustive exercise corroborate that exercise induces free radical production. It has been postulated that free radicals might also damage the sarcoplasmic reticulum resulting in reduced calcium release during depolarisation of the muscle and consequently lead to muscular fatigue. Thus, numerous studies have investigated the efficacy of various antioxidants in ameliorating the deleterious effect of oxidative stress on sports performance. Previous studies which have shown positive effects of antioxidants on skeletal muscle endurance performance were N-acetylcysteine, pycnogenol, quercetin, beetroot juice, cashew apple juice, resveratrol and Montmorency powdered tart cherries. Several studies on the effects of various nutritional supplements with antioxidant properties on endurance performance and antioxidant status have been carried out in our sports science laboratory, School of Medical Sciences and School of Health Sciences, Universiti Sains Malaysia. These supplements include palm vitamin E, caffeine, panax gingseng, *Eurycoma Longifolia Jack* (Tongkat Ali), honey and bee bread. The main findings of these studies will be presented during my talk.

# Abstract: I15: Leisure and Health in Developing Countries: A dialogue with the Sustainable Development Agenda in Brazil

**Published:** 2019-01-01

**Authors:** Ricardo UVINHA

**Affiliations:** Vice Dean School of Arts, Sciences and Humanities Leader of the Interdisciplinary Group of Leisure Studies, Graduate Program in Physical Activity Sciences, University of Sao Paulo (USP), Brazil

The Sustainable Development Agenda (SDGs) of the 2030 Agenda for Sustainable Development was adopted by world leaders in 2015 at the United Nations (UN) Summit. According to the UN, the SGDs are unique as global goals in that they call for action by all countries to promote prosperity while protecting the planet. It was established seventeen goals, being the third one “Good Health and Well-Being” which states that ensuring healthy lives and promoting the well-being for all at all ages is essential to sustainable development. Brazil, the “B” letter of BRICS, is considered a developing country with a strong influence in economic and political sectors in South America. As well as in other countries, non-communicable diseases have increased in Brazil and are the leading cause of death in adults. Prevention and early diagnosis are essential for promoting health and reducing mortality, not only because obesity is a major risk factor for other diseases, but also because it interferes with length and quality of life. In order to encourage physical activity, it is critical to stimulate people to identify physical activities that give them pleasure. A relevant factor for the promotion of regular physical activity is the creation and use of safe public spaces. Safety on the streets is also an essential factor, and this involves urban planning for recreation facilities, bicycle lanes, sidewalks that are in good condition and investments in parks and public facilities. This presentation aims to develop a dialogue with the SDGs, especially the goal regarded to health and well-being, describing several initiatives have already been developed in this regard in Brazil. These include the understanding of leisure as a social right and a vital element for the promotion of health in this country with the creation of bicycle lanes and trails for hiking, revitalization of parks, and street closures at certain times of the day.

# Abstract: I16: The relationship between selected body composition components and self-efficacy among 12–14 year-old rural adolescents in the Eastern Cape Province of South Africa. Purpose of the study

**Published:** 2019-01-01

**Authors:** Maya VAN GENT

**Affiliations:** Associate Professor, Human Movement Science Department, University of Fort Hare, South Africa

Body composition changes drastically in both males and females during the adolescent years. The changes may have a negative effect on their physical health as well as psychological wellbeing, with respect to attributes such as self-efficacy. Being either overweight or obese during adolescence has social, economic and psychological consequences, which include low selfefficacy. Consequently, the aim of this study was to investigate the relationship between anthropometric body composition components and self-efficacy, by conducting a comparison of self-efficacy levels among normal, overweight and obese 12 to 14-year-old rural adolescents in the Eastern Cape Province of South Africa. Overweight and obese boys and girls presented with high levels of self-efficacy, which is in contradiction to other international studies. The results suggest that a positive relationship exists between certain body composition components and self-efficacy. It seems likely that cultural beliefs may exert an influence on the psychosocial perceptions of adolescents in relation to body size, consequently affecting self-efficacy levels. These findings pose a challenge in the efforts of combatting overweight and obesity in adolescents.

# Abstract: I17: Current State of China’s Outstanding Traditional Sports Culture in Curriculum and Teaching of Physical Education in Primary and Secondary Schools

**Published:** 2019-01-01

**Authors:** Xiaozan Wang, Yangtze Dean

**Affiliations:** Youth Scholars of Chinese Ministry of Education, School of Physical Education and Health, East China Normal University, China

This paper aims to promote China’s outstanding traditional sports culture and understand its current state in curriculum and teaching of physical education at primary and secondary schools. A questionnaire survey was conducted on 214 frontline teachers from 212 schools of 18 Chinese provinces and cities; an in-depth interview with 66 teaching and research personnel of physical education, principals and frontline PE teachers across the country; and a field investigation in 6 primary and secondary schools in Henan province. The results show that: 1) various types of traditional sports projects are conducted at schools and 56.54% schools promote traditional sports culture mainly through school clubs or extracurricular activities; 2) teaching staff, quality of teachers, textbooks, sports venues and equipment of traditional sports should be improved; 3) the way of China's outstanding traditional sports culture entering the school should be based on local traditional sports culture; 4) the support of local education policy is a basic guarantee for China’s outstanding traditional sports culture entering curriculum, teaching materials and class; 5) hiring professionals of traditional sports culture to participate in traditional sports activities at school is a temporary and effective way of solving the shortage of teachers; 6) China's outstanding traditional sports must be moderately modified with innovation before it enters the school; 7) the integration of China’s outstanding traditional sports culture is an effective way of inheriting the sports culture; 8) colleges and universities should strengthen the training of traditional sports professionals to improve their ability to inherit and exert their influence to develop traditional sports culture.

# Abstract: I18: Testing Children and Youth Fitness and Sport-Specific Performance

**Published:** 2019-01-01

**Authors:** Erika ZEMKOVÁ

**Affiliations:** Faculty of Physical Education and Sport, Comenius University in Bratislava, Slovakia

Recently, we proposed a Long-Term Sport Diagnostic Model that includes seven age-related stages: Stage 1 (6 to 9 years), Stage 2 (10 to 14 years), Stage 3 (15 to 18 years), Stage 4 (19 to 24 years), Stage 5 (25 to 44 years), Stage 6 (45 to 64 years), and Stage 7 (65+ years). Within this model, we designed tests specifically tailored for these age categories. Targeting young population, our approach to physical fitness testing of school age children was presented at GoFPEP 2014. So far, a variety of tests have been developed for children and adolescents. However, frequently used field tests do not sufficiently reflect various aspects of physical fitness relevant to a particular age and are not sensitive enough to exercise induced changes specific to a particular sport. These traditional methods of assessing the physical fitness of children and adolescents only partially fulfill current needs for testing under sportspecific conditions. Therefore, testing batteries for young individuals should get a makeover. Experience showed that young people participate more intensively and also reach higher exercise goals than with conventional methods when computerized diagnostic and training systems are used. For instance, task-oriented balance tests based on visual feedback control of body position or the agility test performed under simulated competitive conditions seem to be more suitable for children and adolescents than traditional field tests. Both of them are similar to computerized games which may enhance the attention and motivation of children to exercise. Proposed tests for assessment of physical fitness in children and adolescents can be adjusted according to requirements of particular sports and serve as a basis for Sport-Specific Model of Athlete's Performance Testing. Our recent review of testing methods used for the evaluation of the effect of neuromuscular training on sportspecific performance in young athletes will complement the presentation.

# Abstract: K01: Healthy Lifestyle and Physical Activity – A Call for Networking and Collaboration

**Published:** 2019-01-01

**Authors:** Gudrun DOLL-TEPPER

**Affiliations:** Vice-President, German Olympic Sports Confederation Chairperson, German Olympic Academy,Professor of Sport Science/Inclusive Education Department of Education and Psychology, Freie Universitaet Berlin, Germany

Many initiatives and declarations exist at international, national and local levels focussing on the promotion of a healthy lifestyle and increased physical activity over the lifespan. Enormous efforts, undertaken by governments and non-governmental organisations, exist to inform people and to raise awareness about the benefits of a physically active lifestyle and healthy eating. However, despite numerous campaigns and programmes, e.g. by the World Health Organization (WHO), the United Nations Educational, Scientific and Cultural Organization (UNESCO) and many international organisations of physical activity, physical education and sport science, the global problem of a decline in physical activity levels remains and gets even worse. Data exist from different countries around the world which make clear the enormous costs of inactivity affecting individuals and society. It is recommended to increase multiorganisational efforts towards changes in lifestyle and to intensify networking and collaboration at all levels. Selected examples will be presented, e.g. focussing in particular on children and youth, on women, on men, on persons with disabilities and on persons with different cultural backgrounds. Recently, new programmes have been introduced which address people up to 100 years of age and beyond. These initiatives are important in those regions of the world where we notify an increase of life expectancy. Various programmes have also been implemented which offer participation for persons with health issues, such as depression and cancer. In some countries, various forms of collaboration and cooperation amoungst researchers and practitioners from various professional backgrounds exist, which can be used as good examples and an indication for future developments.

# Abstract: K02: Physical Activity and Benefits on Reduction in Chronic Disease

**Published:** 2019-01-01

**Authors:** Stephen KOPECKY

**Affiliations:** Immediate Past President, American Society for Preventive Cardiology, Professor of Medicine, College of Medicine, Mayo Clinic, USA

Lifestyle is now the leading risk factor for early cardiovascular death and chronic disease worldwide, and it is estimated that 80–90% of our health is primarily based on individual lifestyle. Over the past one to two decades, the obesity rate has increased markedly in countries of both upper and lower socio-economic status, including BRICS countries. The two primary components of lifestyle are physical activity and diet. As work requirements push us towards a more sedentary lifestyle, it is critical that we try to maintain regular physical activity, including both moderate frequent daily short episodes to counter our stationary work habits and less frequent episodes of vigorous interval activity. To achieve this, an interdisciplinary approach including teamwork and integration between healthcare providers, exercise physiologists, and dietitians will be required. New healthy food options along with opportunities for safe physical activity will be essential. The Lifestyle and Sports Medicine fields must be positioned to lead this lifestyle transformation via population, government, and industry education.

# Abstract: K03: Kinderkinetics, Physical Activity and Health: A South African Perspective

**Published:** 2019-01-01

**Authors:** Anita Pienaar

**Affiliations:** Chair: Kinderkinetics, South African Professional Institute for Kinderkinetics (SAPIK), School of Human Movement Sciences, North-West University, South Africa

This presentation will focus on Kinderkinetics as a specialized health profession that are used in a South Africa context to address the challenges of physical inactivity among children in support of the exercise-as-medicine preventative belief. Over many years South Africa had major challenges due to various reasons to address the physical activity needs and the subsequent health problems of South African children growing up in very diverse circumstances. This include taking Physical Education as a subject out of the School curriculum in 1996, which at that time created a big void in expertise to address, not only the motor and physical developmental needs of young children, but also the problems that arise as a result of physical inactivity. This as well as a need towards creating new avenues that can create job opportunities to address work shortages in our country, provided a much needed impetus towards the developing of this new curriculum where we started to train health practitioners to become specialists that can address the improvement of health and well-being of children, also on an entrepreneurial level, based on physical activity. This journey towards developing this professional field and where it stands today, will be discussed. However, although scientific evidence of the importance of sound motor and physical development to the health and well-being of children, also on a cognitive level, are well documented, it is also important to provide evidence based research but also practice based evidence as proof of successful interventions. Research findings from within the Kinderkinetics program that can corroborate that the delivering of different Kinderkinetics programs has scientific merit in a school, clinic, therapeutic and sport environment, will also be discussed.

# Abstract: O01: The impact of a multiple health behaviour intervention strategy on the prevalence of coronary artery disease risk factors, health-related physical fitness, functional occupational performance, health-risk behaviours, and work-related musculoskeletal disorders among municipal firefighters

**Published:** 2019-01-01

**Authors:** G Achmat, L Leach

**Affiliations:** 1 Department of Sport Recreation and Exercise Science University of the Western Cape. Republic of South Africa

## Introduction

There is a compelling body of evidence that coronary artery disease (CAD) risk factors are present in people of all ages. The extent to which the problem exists among firefighters in South Africa (SA) has not been confirmed in the literature.

## Aim

This study aims to assess the impact of a multiple health behaviour intervention strategy on the coronary artery disease (CAD) risk factors, health-related physical fitness (HRPF), functional occupational performance (FOP), health-risk behaviours (HRBs), and work-related musculoskeletal disorders (WMSDs) among municipal firefighters in the City of Cape Town Fire and Rescue Service (CCTFRS).

## Methods

An experimental study design will be used, wherein 212 participants will be randomly sampled and have baseline tests measured. Thereafter, the participants will be randomized into four groups consisting of 54 firefighters per group, both males and females, i.e., one control group (CG), one health information (HIG), one physical activity group (PAG), and a multiple treatment group (MTG). The groups will be tested before and after a 12-week multifactorial intervention strategy. The intervention will be based upon Prochaska’s Transtheoretical Model of behaviour change.

## Data Collection

Participant information will be obtained using validated self-report questionnaires, namely, the physical activity readiness questionnaire (PAR-Q), the stages of readiness to change questionnaire (SRCQ), the international physical activity questionnaire (IPAQ), the Adult COMBO Health Behaviour Measures Questionnaire (ACBMQ) and the Nordic Musculoskeletal Questionnaire (MSQ). In addition, various physical and hematological (blood) measurements will be taken. The following measurements will be taken before and after the intervention programme: 1) Coronary artery disease risk factors, namely, hypertension, obesity, fasting blood cholesterol and fasting blood glucose; 2) Health-related physical fitness, namely, body composition, cardiovascular fitness, muscular strength, muscular endurance, and flexibility; and 3) Functional occupational performance (six firefighting-specific tasks). Furthermore, the following health-risk behaviours will be recorded, namely: physical activity, nutrition, managing stress, avoiding destructive habits, practising safe sex, adopting safety habits, knowing first aid, personal health habits, using medical advice, being an informed consumer, protecting the environment and mental well-being, as well as various work-related musculoskeletal disorders. Where the outcome variables are normally distributed, the groups will be compared using repeated-measures ANOVA. For outcomes with a non-normal distribution or ordinal level data, the nonparametric Wilcoxon Rank Sum test will be used for group comparisons. For nonparametric methods, analysis will be done using Cochran-Mantel-Haenszel (CMH) methodology, stratifying on the baseline values. For nominal level outcomes, groups will be compared by Chi-square tests for homogeneity of proportions. Where baseline values are needed to be incorporated into the analysis, this will be done using CMH methodology. A significance level p < 0.05 will be used to indicate statistical significance.

## Ethical Considerations

Ethics clearance will be requested from the Biomedical Research Committee at the University of the Western Cape, and permission will be requested from the Chief Fire Officer of the City of Cape Town Fire and Rescue Service. Participants will be informed about the nature and the scope of the study, including testing procedures, risks and benefits, prior to testing. Participation in the study will be voluntary, and participants will have the choice to withdraw at any time without any negative consequences. Data will be stored in a locked filing cabinet in the supervisor’s office, and electronic data on a password-protected laptop. All data will be stored for a period of five years before being destroyed.

# Abstract: O02: Training load and quality of recovery in Ethiopian higher league football players

**Published:** 2019-01-01

**Authors:** Fikreyesus Daniel Adula, van Heerden Johan

**Affiliations:** 1 Wachemo University, Ethiopia; 2 Department of Biokinetics, Exercise and Leisure Science, University of KwaZulu-Natal, South Africa

## Introduction

It has been suggested that player fitness and the quality of training offered by coaches could pose a barrier to the performance standard in Ethiopian football. There is no research-based approach to the methods used to monitor maximum and minimum intensity and volume of training imposed on the players and their recovery during the season. The purpose of this study was thus to monitor training load and recovery among football players in the Ethiopian Higher League over a pre-season and in-season period of the competition.

## Methods

A squad of twenty-five male footballers (age 22.08 ± 1.41 years, weight 70.24 ± 6.45 kg, height 175.60 ± 7.01cm, BMI 22.78 ± 1.77) playing for a professional team in the Ethiopian Higher Football League were tracked over a macrocycle of the season comprising ten weeks. Rating of Perceived Exertion (RPE) and Total Quality of Recovery (TQR) scores were recorded for 40 days. Session training load (sTL) was subsequently calculated as the product of session Rated Perceived Exertion (sRPE) and session training duration (sTD) in Arbitrary Units (AU).

## Results

Similar perceived exertion mean values of 11.62 ± 1.56 and 12.20 ± 2.34 RPE (p>0.05) corresponding with ‘fairly light’ and ‘somewhat hard’, were recorded in preseason and in-season, respectively with ‘poor recovery’ similar ‘poor’ TQR recovery recorded for both the pre-season (11.09 ± 0.64) and in-season (11.2 ± 1.53). A higher mean sTL incorporating duration of sessions was registered for the in-season (1009.34 ± 193.28 AU (p≤0.0001) than for the pre-season (887.3 ± 117.09 AU). Variations across the in-season showed a decrease (p≤0.05) in sTL from 3009.6 ± 750.69AU at the beginning (week 3) to the middle (week 6) with 2610.0 ± 717.86AU followed by an increase (p≤0.05) to 23369.6 ± 1773.99AU at the end (week 10) of the season. Corresponding perceived exertion and recovery at week 3 was somewhat hard (RPE 12.53 ± 3.10) and poor (TQR 10.44 ± 3.1) followed by a less taxing fairly light (RPE 10.97 ± 3.08) but poor recovery (TQR 10.31 ± 3.30) at week 6, and at week 10 perceived exertion was higher, but still approximating fairly light (RPE 11.36 ± 4.80) and recovery scores improved (p>0.05) slightly (TQR 11.39 ± 3.11) showing a matched perceived intensity of training and the degree of recovery. Over the in-season, compared to week 3 and 4, a higher mean sTL was recorded in week 5 (4039.2 ± 193.28 AU (p≤0.0001) followed by a reduction in week 6 (2610.0 ± 717.86 AU (p≤0.0001) and another increase at the end of the season in week 9 and 10 (3369.6 ± 1773.99 AU (p≤0.0001).

## Conclusions

Monitoring the sessional training loads over the pre- and in season macrocycle showed evidence of periodization in the form of cyclic progression with respect to higher and lower volumes of training. Corresponding perceptions of improved recovery during lighter phases of training were, however, not evident and requires further analysis to identify the specific aspects of fatigue experienced.

# Abstract: O03:The effect of kinesio taping and low energy laser application in myofascial pain syndrome

**Published:** 2019-01-01

**Authors:** Balci L Ataş, NB Büyükavcı, S Yağmur, M Alkan, Ş Alkan

**Affiliations:** 1 Department of Physiotherapy and Rehabilitation, Bahçeşehir University, İstanbul, Turkey; 2 Department of Physiotherapy, Bahçeşehir University, İstanbul, Turkey; 3 Institute of Health Sciences, Bahçeşehir University, İstanbul, Turkey

## Introduction

Myofascial pain syndrome (MPS) associated with active myofascial trigger points is a common problem can cause pain, muscle spasm, sensitivity, weakness, limitation of range of motion in the patients. Physical therapy modalities and exercise are most commonly used in the treatment of MPS. However the efficacy of low level laser therapy (LLLT) and kinesio taping on patients with MPS have not been investigated. The purpose of this study was to determine the effects of kinesio taping (KT) and LLLT on pain intensity, sleep quality and quality of life in the patients with MPS.

## Method

Forty-five volunteers (33 Male; 11 Female; mean age: 22.24±1.13 years) with MPS of the upper trapezius muscle were recruited. Participants were divided into 3 groups which consisted of i) LLLT group (LLLT+exercise application) ii) KT group (KT+LLLT+exercise application) iii) an exercise group. LLLT group received laser therapy once a week in a 4-week of treatment period. KT group received kinesio taping for the upper trapezius muscle in addition to LLLT application. Pain intensity, sleep quality and quality of life were assessed by Visual Analogue Scale (VAS), Pittsburgh Sleep Quality Index (PSQI) and Short Form-12 (SF-12) respectively at the onset and at the end of four weeks of training period.

## Results

Pain intensity significantly improved following the 3 interventions (p<0.05). However, the changes in the VAS of the KT group were significantly greater than those of the other 2 groups (p<0.05). There were statistically significant changes in the physical and mental functioning items of the SF-12 scores in the KT group (p<0.05). However, a statistically significant change was found only in the physical function items of the SF-12 in the LLLT group (p<0.05). Additionally, comparisons of changes in the SF-12 scores among the 3 groups revealed no significant differences (p>0.05). PSQI scores significantly improved in the KT and exercise groups. However, no statistically significant changes were found in in the PSQI scores of the LLLT group (p>0.05).

## Conclusion

Our findings suggests that adding KT application to laser therapy may be effective for pain relief and increasing sleep quality and quality of life in patients with MPS.

# Abstract: O04: The relationship between nomophobia and physical activity among students in Turkey

**Published:** 2019-01-01

**Authors:** L Atas Ataş Balcı

**Affiliations:** 1 Department of Physiotherapy and Rehabilitation, Bahçeşehir University, İstanbul, Turkey

## Introduction

The increasing use of new technologies involving personal computers, tablets and mobile phones are leading to changes in the habits and behavior of young adults. The problematic and excessive use of technology may have some negative effects such as nomophobia. Nomophobia (No mobile phone phobia) refers to the fear and anxiety of not being able to access a mobile phone/smart phone or not being able to communicate through a mobile device. Nomophobia and excessive usage of smart phones may lead to depression, low academic achievement, decreased of productivity and impairment of social and emotional functioning. Moreover, nomophobia can be associated with physical activity, but the relationship between nomophobia and physical activity is not known. Therefore the aim of this study is to determine the relationship between nomophobia and physical activity status of students.

## Method

The Turkish version of the Nomophobia Questionnaire (NMQ) and International Physical Activity Questionnaire (IPAQ) were used to evaluate nomophobia and physical activity status of 137 students (84 Female; 53 Male; mean age: 22.10±1.80 years; BMI: 23.43±4.18) in the Department of Physiotherapy and Rehabilitation at Bahceşehir University, in Istanbul in Turkey. Spearman’s correlation was carried out to determine the relationship between NMQ total scores and IPAQ total scores.

## Results

The mean total NMQ scores and the mean total IPAQ scores of the participants were 86.15± 30.86 and 2342.78±1807.49 respectively. Most of the students were found to have severe nomophobia (48.2%). The results showed that, female students have a higher tendency to show nomophobic behaviors compared to male students (p=0.04) There was a statistically significant strong negative relationship between NMQ total scores and IPAQ total scores (r= −0.541; p=0.000). Also, there was statistically significant moderate positive relationship between BMI results and NMQ total scores (r=0.168; p=0.05).

## Conclusion

The lower the level of physical activity, the higher the risk of exhibiting nomophobic behaviors. Additionally; the current study indicated that the students with higher level of BMI tend to show more nomophobic behaviors.

# Abstract: O05: Training loads and injury profiles in elite South African rugby players

**Published:** 2019-01-01

**Authors:** Curt Barnes, Theresa Burgess, Kim Buchholtz

**Affiliations:** 1 Division of Physiotherapy, Department of Health and Rehabilitation, University of Cape Town, South Africa; 2 Division of Exercise Science and Sports Physiotherapy, Department of Human Biology, University of Cape Town, South Africa

## Introduction

Across professional team sports, professional Rugby Union has one of the highest reported incidence of injury. Despite the inherent risk of injury due to the high physical demands, training load in Rugby Union is an independent risk factor to injury. The Super Rugby tournament is a platform to further investigate injury and training load patterns within Rugby Union. The nature of the Super Rugby tournament has been associated with high rates of injury, but the relationship between training loads on injury profiles are unclear.

## Methods

A descriptive, observational, surveillance study design over twenty-eight weeks. Thirty-nine adult participants were recruited from one South African team over pre-season, early and late competition of the 2017 Super Rugby tournament. Routinely collected data by the squads medical personnel was collated. Training load data included squad size, training or match day, the duration of training or matches, and internal and external training load measures for training and matches. Injury data included the participants age, the injury counts, the type of injury, the main and specific anatomical location, and the mechanism and severity of injury.

## Results

The overall incidence of injury was 12.8 per 1000 player hours. The majority (48.8%) of injuries occurred in the early competition phase. A significant negative correlation between injury and internal training loads were detected in the preseason phase (r = −0.34, p = 0.03). No significant correlations were detected between external training load and injury. Odds ratios were used to assess the associations between internal and external acute to chronic ratios, and injury. No significant relationships were found.

## Conclusion

The incidence of match injuries in this study was significantly higher than previously reported incidence rates in the Super Rugby tournament. The profiles of match and training injuries, anatomical location, type, mechanism and severity of injuries are similar to previous studies. Internal training load and injury were significantly correlated in the preseason phase. Further studies are required to determine the relationship of training loads on injury profile over consecutive seasons and in multiple teams.

# Abstract: O06: Injury profiles and illness rates in elite South African rugby players

**Published:** 2019-01-01

**Authors:** Curt Barnes, Theresa Burgess, Kim Buchholtz

**Affiliations:** 1 Division of Physiotherapy, Department of Health and Rehabilitation, University of Cape Town, South Africa; 2 Division of Exercise Science and Sports Medicine, Department of Human Biology, University of Cape Town, South Africa

## Introduction

Professional Rugby Union is a popular international team sport and is known to have one of the highest reported incidences of injury and illness across sporting codes. The Super Rugby tournament is played annually between professional Rugby Union teams and is one of the most competitive sports tournaments in the world. The demanding nature of the tournament has been associated with high rates of injury and illness. Epidemiological data on injury profiles and illness patterns during these professional tournaments are an essential first step in the injury prevention process.

## Methods

A descriptive, observational, surveillance study design was conducted during the 2017 Super Rugby tournament. Thirty-nine adult participants were recruited from one South African franchise over a complete season of twenty-eight weeks, including preseason, early and late competition. Data were collected routinely by the team medical personnel. Descriptive and injury data included the participants age, the injury counts, the type of injury, the specific anatomical location, and the mechanism and severity of injury. Illness data included illness counts, the bodily system affected, symptoms and cause of illness, the specific diagnosis and time-loss.

## Results

The overall incidence of injury was 12.8 per 1000 player hours. The majority (48.8%) during the early competition phase. The incidence of match injuries (241.0 per 1000 player hours) was significantly higher than training injuries (3.3 per 1000 player hours). The lower limb sustained the greatest proportion of injuries (62.5%). Muscle or tendon injuries accounted for 64.9% of all injuries. The tackle accounted for 28.8% of all injuries and 37.5% of all injuries were of a ‘moderate’ severity. The proportion of players that sustained a time-loss injury was 76.9% (n = 30). The proportion of players that sustained a time-loss injury severe enough to prevent eight days or more of participation in training or matches was 25.6% (n = 10). The overall incidence of illness was 1.8 per 1000 player days. Twenty-eight percent of players (n=11) acquired an illness. Acute respiratory tract infections (28.6%) was the most common diagnosis and the majority of illnesses (64.3%) did not result in time-loss.

## Conclusion

The incidence of match injuries in this study was significantly higher than previously reported incidence rates in the Super Rugby tournament. The profiles of match and training injuries, anatomical location, type, mechanism and severity of injuries are similar to previous studies. Illness rates were significantly lower than reported in previous studies. Further studies are required to determine the injury profiles and illness rates over consecutive seasons and in multiple teams.

# Abstract: O07: Prevalence and type of injuries in South African trail runners

**Published:** 2019-01-01

**Authors:** Susan H. Bassett

**Affiliations:** Harry Van Wyk Steyn Swart

## Introduction

Trail running is becoming an ever more popular sport worldwide with the trail running population having more than doubled since 2006. More and more people are moving from the monotonous and repetitive running nature of road running to the trails. With the popularity of trail running increasing, so are the number of injuries being reported. Injuries range from acute to chronic and include muscular, skeletal, ligamentous and tendinous. The amount of time an athlete cannot train or compete due to injury depends on its severity. This study aims to investigate the prevalence and type of injuries of trail runners in South African trail runners aged between 19–40 years.

## Methods

- This study will employ a descriptive, survey research design involving the use of a questionnaire sent to various trail running groups or clubs, which will be used to collect specific data from the runners. The sample will include male and female trail runners from the ages of 19–40 years from both recreational to elite level, who have at least 2 years of running experience. An information sheet, explaining the study and what the participant will be asked to do, will be sent via email to various trail running groups and clubs across the country to distribute to their members. Participation will be purely voluntary and a link will be provided, via the running club, to the Google Form questionnaire. By completing the questionnaire, consent will be implied. The sample size is dependent on the amount of respondents. Descriptive statistics will be utilized to analyse the data. In doing so, the type of injury and prevalence thereof will be established.

## Results

This study data has not be collected yet, but we aim to do this by August this year, so the study will be complete in time for presentation at the conference in October.

# Abstract: O08: The effect of a three month Hopsports brain breaks® intervention programme on the attitudes of grade 6-learners towards physical activity and fitness

**Published:** 2019-01-01

**Authors:** Jacqueline Bonnema, Dané Coetzee, Anita Lennox

**Affiliations:** 1 Physical Activity, Sport and Recreation, Faculty of Health Sciences (PhASRec) focus area, North-West University (Potchefstroom Campus), South Africa

## Introduction

Negative attitudes towards physical activity (PA) have been reported to have an influence on the PA levels of children and the choices they make to be physically active or not. Research also indicated that children’s PA levels are more prevalent to decline between the ages of 12- to 15-years. This study investigated the effect of a three month HOPSports Brain BreakR intervention programme on children’s attitudes towards PA and fitness.

## Method

Attitudes towards PA were assessed by means of the Attitudes towards Physical Activity Scale (APAS) questionnaire in children between the ages of 11- to 12-years. The experimental group consisted of 75 children (44 boys and 31 girls) with a mean age 11.4 years (±0.54) and 39 children (12 boys and 27 girls) for the control group with a mean age of 11.71 years (± 0.49).

## Results

The results indicated that there were no significant differences between the two group’s attitudes towards PA and fitness with the pre-test. However, after the intervention programme there was a significant difference with a large effect size (d ≥ 0.8) which includes the attitudes towards the benefits of PA (α = .70; d = 1.13), importance of PA (α = .65; d = 0.63), self-perception of physical fitness (α = .86; d = 0.77) and their attitudes towards support in the environment and their interest for PA (α = .83; d = 1.25).

## Conclusion

Recommendations from this study are that more focus should be on 12-year old children because the decline in PA levels is more prevalent and their attitudes are more influential as well as to create opportunities where children can be equally active.

# Abstract: O09: Workload does not impact performance responses in the Indian Premier League cricket

**Published:** 2019-01-01

**Authors:** Lee Pote, Devon Barnard, Catherine Munro, Dave Goble, Belinda Campbell, Candice Christie

**Affiliations:** Human Kinetics and Ergonomics, Rhodes University, Grahamstown, South Africa. Movement Physiology Research Laboratory, School of Physiology, Wits University, Johannesburg, South Africa

## Introduction

Elite cricket is an international sport dominated by three main formats, namely test, one day and most recently, T20 matches; each with its own workload requirements. The performance aspect of the game is of particular importance in bowlers, who are essential to a team’s success as they restrict the opposition’s runs and attempt to bowl them out. As a result, better bowling performance results in improved overall team performance and an increased chance of winning. Research has shown that bowling requires players to perform multiple quick actions simultaneously, which can increase the physical demands placed on the body. Thus, an increase in workload may have an effect on a player’s bowling performance; which is of a particular concern with an increase in the amount of competitions that are currently being played globally. The Indian Premier League started in 2008 and has been ongoing since then, with the prize money for the winning team in 2018 being four million dollars. The aim of this investigation was to determine the relationship between bowling performance and player workload in elite Indian Premier League bowlers.

## Methods

The sample consisted of eleven male, professional cricket bowlers (mean ±SD, 27 ± 5.61 years; stature: 1.77 ± 0.07 m; mass: 72.09 ± 10.89 kg) currently playing for a team in the Indian Premier League cricket competition. Both bowling disciplines (seam and spin) as well as all-rounders were considered for the investigation. Workload data was obtained using Microzone data capturing software and was reduced into total weekly workload and total cumulative workload. The match was gathered from ESPN CricInfo. Match data included; overs bowled, runs conceded, wickets taken, bowling economy, dot balls bowled, 4's conceded, 6's conceded, wides bowled. The performance data and match data were used to establish relationships between workloads and performance related data.

## Results

No significant relationships were found between total weekly workload and performance. Conversely, cumulative workload did have an impact on some performance measures. Specifically, the total cumulative workload of weeks 1 to 4 had a strong negative association (r = 0.89) with total overs bowled in the fourth match week (p=0.041). In addition, total cumulative workload of weeks 1 to 7 had a strong positive (r = 0.83) relationship with the total number of 6’s conceded in match week 7. No other relationships were observed.

## Conclusion

The most important finding of the investigation is that workload does not predict performance in this Indian Premier League cohort of bowlers. However, associations were observed between total cumulative workload and specific measures. This may be due to the fact that the nature of the game of cricket is exceptionally unpredictable and t is challenging to measure performance related responses.

# Abstract: O10: Influences of early perceptual-motor proficiency, gender, overweight and socioeconomic status on academic achievement of primary school children: longitudinal data of the north-west child study

**Published:** 2019-01-01

**Authors:** E De Waal, AE Pienaar, SM Ellis

**Affiliations:** 1 Physical Activity, Sport and Recreation (PhaSRec), Focus area, Faculty of Health Sciences, Potchefstroom Campus, North-West University, Potchefstroom, Republic of South Africa; 2 Unit for Business Mathematics and Informatics, North-West University, Potchefstroom, Republic of South Africa

## Introduction

Perceptual-motor proficiency and academic achievement is considered to be fundamentally intertwined. Excessive body weight might provide and additional burden to this reported link, while socio-economic status (SES) and gender are also reported to influence academic achievement. This study aimed to determine how perceptual-motor proficiency, gender, overweight and SES are related to academic achievement longitudinally.

## Method

The study included 381 participants (181 boys; 200 girls, 168 high SES; 218 low SES) that formed part of the NW-CHILD longitudinal study covering the primary school period of 7 school years, starting at grade 1 (6.86 years, ± 0.39) in 2010 until grade 7 (12.9 years, ±0.38) in 2016. The *Bruininks Oseretsky Test of Motor Proficiency-2 (BOT-2) Short Form* and selected composites were used to assess perceptual-motor proficiency at baseline and at two follow-up time-points, while provincial and mid-year academic school reports determined academic achievement.

## Results

Correlation analyses confirmed significant moderate (*r* = .19 to *r* = .36) correlations between perceptual-motor proficiency at early and older ages and academic achievement. Normal weight children demonstrated practically significant better strength, balance and running speed and agility. A latent growth curve analysis for academic achievement within Structural Equation modelling (SEM) were tested, and indicated prior academic achievement, socio-economic status and gender as the strongest long-term determinants, while persistent overweight/obesity did not influence academic achievement significantly. Perceptual-motor proficiency correlated with grade 1 academic achievement and showed an indirect relationship to grade 7 academic achievements via academic performance in grade 4. Academic achievement in grade 1 and SES had the largest effect on both academic achievements in grade 4 and in grade 7, according to the standardised regression weights and total effects. Boys and participants with lower SES had lower academic achievement at ages 9 and 12.

## Conclusion

Influences that should be addressed timeously include academic achievement in grade 1 and SES, which had the largest effect on both academic achievement and perceptual-motor proficiency, especially in the earlier grades.

# Abstract: O11: A nine-week pilates exercise programme reduces pain in the upper body of female office workers

**Published:** 2019-01-01

**Authors:** Kimberly-Ann Decina, Paola S Wood, Steve Olorunju

**Affiliations:** 1 Department of Physiology, Faculty of Health Sciences, University of Pretoria, Pretoria 0002, South Africa; 2 Biostatistics Unit, South African Medical Research Council, Private Bag X385 Pretoria 0001, South Africa

## Introduction

The increase of pain prevalence in the upper body of female office workers due to prolonged visual display unit (VDU) work is a growing socioeconomic concern amongst employers. Associated risk factors include poor posture, static muscle contractions and being female. Pilates focuses on improving body awareness and movement and has shown to be useful in the treatment of chronic lower back pain. Thus, the study aimed to determine the effects of the Pilates method of exercise on pain and functionality of the neck, shoulders and/or upper back in female office workers in Pretoria.

## Methods

An experimental, pre-test post-test control group design was used. Female office workers between 30–55 years (n=60) performing ≥4hrs of VDU work per day with pain in their neck, shoulders and/or upper back, volunteered for the study. Participants were randomly assigned to a control group (CG) (n=30) or experimental group (EG) (n=30), using the fishbowl technique. The EG completed 18 Pilates sessions over nine weeks. Baseline and post-intervention measurements included pain prevalence (Nordic Musculoskeletal Questionnaire); pain intensity (Visual Analogue Scale); scapular stability (lateral scapular slide test), neck and shoulder range of motion (ROM) and shoulder strength (MicroFET3 digital inclinometer and dynamometer). Statistical analysis included paired t-tests, unpaired t-tests and effect sizes.

## Results

At post-test the EG reported a reduction in pain prevalence in the neck (46.67%, p<0.001), shoulders (66.67%, p<0.001) and upper back (33.33%, p=0.002). Baseline pain intensity values averaged at 5.4, with a significant 33.3% (p<0.001) decrease following the intervention. The most restricted ROMs were neck rotation, neck lateral flexion, shoulder flexion, shoulder abduction and shoulder internal rotation (IR). Significant post-intervention improvements were noted in neck flexion (p=0.008; d=0.07), neck extension (p=0.004; d=0.13), neck lateral flexion (p<0.001; d=0.75 left, d=0.90 right), neck rotation to the right (p=0.014; d=0.59), shoulder extension (d=1.03 left, d=1.01 right), left shoulder flexion (p=0.001; d=0.33), shoulder abduction (p=0.013 left; d=0.91 left, d=0.77 right) and left shoulder external rotation (ER) (d=0.62). Shoulder flexion, extension and abduction strength values were below the norms at baseline. The Pilates intervention significantly increased left shoulder flexion (p=0.048; d=0.18), shoulder extension (d=0.82 left, d=0.89 right), right shoulder abduction (p=0.018; d=0.12), shoulder IR (d=0.79 left, d=0.84 right), shoulder ER (p=0.010 left; d=1.36 left, d=0.45 right). Scapular instability at baseline improved significantly (p<0.001) at post-test.

## Conclusions

The Pilates intervention successfully reduced upper body pain prevalence and intensity; increased neck and shoulder ROM and shoulder strength with variable clinical significance; and improved scapular stability. Evidence supports the Pilates method of exercise to be included in corporate wellness programmes.

# Abstract: O12: Tackle and ruck technique proficiency within academy and senior club rugby union

**Published:** 2019-01-01

**Authors:** S den Hollander, M Lambert, B Jones, S Hendricks

**Affiliations:** 1 Division of Exercise Science and Sports Medicine, Department of Human Biology, Faculty of Health Sciences, the University of Cape Town and the Sports Science Institute of South Africa, Cape Town, South Africa; 2 Health through Physical Activity, Lifestyle and Sport Research Centre (HPALS), Faculty of Health Sciences, the University of Cape Town, South Africa; 3 Western Province Rugby Academy, Cape Town, South Africa; 4 Carnegie Applied Rugby Research (CARR) Centre, Institute for Sport, Physical Activity and Leisure, Leeds Beckett University, Leeds, UK; 5 Yorkshire Carnegie Rugby Union Football Club, Leeds, UK; 6 Leeds Rhinos Rugby League Club, Leeds, UK; 7 England Performance Unit, The Rugby Football League, Leeds, UK; 8 School of Science and Technology, University of New England, NSW, Australia

## Introduction

This study examined the validity of a tool that assesses tackle and ruck technique in training, and established reference data for tackle, ball-carry and ruck technique at different levels of play in rugby union.

## Methods

One hundred and thirty-one amateur rugby union players; 37 senior, 51 first grade academy and 43 second grade academy players, participated in a two-on-two contact drill. The drill was filmed and the players’ tackle, ball-carry and ruck technique were assessed using standardized technical criteria. One-way analysis of variance was used to test for differences between the levels (*senior, academy 1^st^ and academy 2^nd^*) and Cohen’s Effect Sizes were used to calculate the magnitude of these differences.

## Results

*Senior* level players scored significantly higher in all three assessments; tackle technique *senior* vs *academy 1^st^* (p<0.01, ES=0.7, moderate), senior vs *academy 2^nd^* (p<0.01, ES=0.7, moderate); ball-carry technique *senior* vs *academy 1^st^* (p<0.01, ES=0.6, moderate), *senior* vs *academy 2^nd^* (p<0.01, ES=0.8, moderate); ruck technique *senior* vs *academy 1^st^* (p<0.01, ES=0.7, moderate), *senior* vs *academy 2^nd^* (p<0.01, ES=0.4, small).

## Discussion

These findings emphasize the importance of developing contact technique to allow players to progress to higher levels, and provide validity to an assessment tool which can facilitate this process.

# Abstract: O13: Associations of physical activity levels and engaging in substance use and other mental health risks among american youth

**Published:** 2019-01-01

**Authors:** Kele Ding, Jiangzhen Yang

**Affiliations:** 1 School of Health Science, Kent State University, Kent, Ohio, USA; 2 Center for Injury Research and Policy, Dept. of Pediatrics, College of Medicine, The Ohio State University, Columbus, Ohio, USA

## Introduction

Regular physical activity has been recognized as an effective strategy in reducing the risk of many adverse health consequences. The benefits of physical activity significantly increase through higher intensity, greater frequency, and/or longer duration of physical activity. However, whether individuals who are physically active are more likely to engage in other health-related behaviors remain unclear. The purpose of this study is to investigate whether physically active youth also engage more in other health-related behaviors.

## Method

The Youth Risk Behavior Surveillance System (YRBSS) combined data set was used as a basic data source. Physical activity level was defined by a question asking about the number of days in past 7 days prior to the survey that youth has engaged in a total of 60 minutes or more of physical activity. The responses on physical activity level were categorized into three comparison groups: 1) 5 or more days a week, 2) one to four days a week, or 3) none. A series of substance use and mental health disorders were selected for examination. Presence of each of these behaviors/disorders was treated as a dichotomous variable. Sampling unit was used as cluster variable, and population weight score was used to weight each case. Generalized Linear Modeling with GEE adjustment was used to test the odds of each behavior among the physical activity categories. Sex, age, race, grade, and GPA were included in the analysis as control variables. Significance was defined at p < 0.05.

## Results

A total of 14,238 youth who answered the physical activity question on the 2017 YRBSS were included, while 527 with missing values were excluded. Of 14,238 youth, 7,329 (51.5%) were females and 6,789 (47.7%) were males, with an average age of 16 (ranging from 12 to 18 years). More than 40% (43.8%) of youth reported 5–7 days per week of physical activity for 60 min or longer, followed by 39.3% of youth who reported one to four days per week of physical activity. However, 16.9% of youth reported 0 days of physical activity in the past week. After adjusting for demographic variables, youth who were more physically active tended to have higher odds of engaging in the risk behaviors. Significantly higher odds were found for tobacco use, steroid use, and marijuana use; drinking driving or riding with a drunken driver. Youth who were physically active were less likely to experience feelings of sadness or hopelessness, and less likely to attempt suicide.

## Conclusion

Physical activity is recommended for youth. However, less than half of youth in the study sample met the recommended activity level. Relatively, youth who were more physically active had higher odds of engaging in drug use behaviors but lower odds of reporting mental health-related disorders. The results of this study suggest the importance of promoting mental health-related behaviors while promoting physical activity among youth.

# Abstract: O14: End of season evaluation of coaches by athletes (ESECA) of the University of the Philippines

**Published:** 2019-01-01

**Authors:** R Dizer, OYS Santelices

**Affiliations:** 1 Department of Physical Education University of the Philippines, Diliman, Quezon City, Philippines; 2 Department of Sport Science, University of the Philippines, Diliman, Quezon City, Philippines

## Introduction

Sports Coaching is a complex job that it needs to look into the many details involved in this profession rather than to use these outcomes in competitions to measure and evaluate coaching performance. The aim of the research is to construct an instrument that would enable coaches identify their self-awareness of their own coaching and teaching skills as perceived by the athletes in 29 collegiate sports events with 3 performing dance groups under the University of the Philippines’ Varsity Sports Program.

## Methods

This test development utilized test construction methodology to arrive at its objective in coming up with the End of Season Evaluation of Coaches by Athletes (ESECA) consisting of the use of Likert Scale and open-ended questions. The researchers did expert validation by getting experts in test construction from the College of Social Science and Philosophy of the University of the Philippines. Based from their comments and suggestions, the researchers did item analysis to come up with a validated through face validity.

## Results

These procedures resulted in the construction of the initial draft of the End of Season Evaluation of Coaches by Athletes (ESECA) with the following concepts: 1. Coaching Principles/Philosophy, 2. Organization/Administration, 3. Coaching Methodology, and 4. Overall Evaluation. Comments and suggestions were also included in order to have a subjective evaluation of the coaches.

## Conclusions

With the ESECA, this can already be used as a pilot study by administering it to the athletes of the University of the Philippines to evaluate the coaches of their respective sports. And future research can be done if there is a need to improve the ESECA.

# Abstract: O15: Teacher perceptions of learner motivation after the completion of a physical education in-service training programme

**Published:** 2019-01-01

**Authors:** D Du Toit, N Van der Merwe, V Oelofse

**Affiliations:** 1 Physical Activity, Sport and Recreation (PhASRec) Research Focus Area, Faculty of Education, North-West University, South Africa; 2 Physical Activity, Sport and Recreation (PhASRec) Research Focus Area, Faculty of Education, North-West University, South Africa; 3 Faculty of Education, North-West University, South Africa

## Introduction

The shortage of trained and qualified Physical Education (PE) teachers in South Africa is worrying, in view of the decline in physical activity and the associated increase in health risks among South African children, which can be addressed by effective PE. The competence and effectiveness of the PE-teacher can also have a positive effect on the motivation levels of learners to participate in PE and physical activities. In-service training programmes aiming to improve the competence, effectiveness and needs-support skills of PE teachers, can therefore indirectly enhance the motivation levels of learners in the PE class. The purpose of this study was to investigate the effect of an in-service training programme on learner motivation, as perceived by their teachers.

## Methods

The study was based on qualitative research within the theoretical framework of the Self Determination Theory (SDT) of Deci and Ryan (2000), which postulates the satisfaction of the basic psychological needs of autonomy, competence and relatedness for determining intrinsic motivation. Individual interviews were conducted with eight purposefully chosen participants in a five-day, in-service PE teacher training programme, on the last day of the programme and four months later. Qualitative data were also collected from open-ended questions, and supported by limited quantitative data from questionnaires. Categories and themes were identified and interpreted according to specific steps and guidelines, while the quantitative data were analysed using descriptive statistics.

## Results

The first theme that emerged strongly from the qualitative data reflected that the participants experienced higher levels of learner motivation after the training programme. The reasons for these improved motivation levels were categorized as four subthemes, all relating to the psychological needs of the learners. Firstly, the teachers ascribed their learners’ improved motivation to their implementation of new and creative ideas for practical activities. Secondly, learner motivation was influenced by the higher levels of self-confidence, competence and motivation of their teachers, which strengthened the learners’ perceptions and understanding of the value of PE. Thirdly, according to the participants, the discouraging impact of too few or no PE equipment was overcome by their newly acquired knowledge and skills to improvise apparatus. The participants further felt that their improved competence and effectiveness to meet the needs of large and diverse PE classes, impacted on learner motivation, and that all PE teachers should receive specialized training in this regard. The second theme involved barriers that still impacted negatively on learner motivation, which also included large and diverse classes, unqualified and unenthusiastic teachers, a lack of support from principals and colleagues, and poor facilities. The quantitative results supported the above findings.

## Conclusions

According to the findings, the in-service training programme contributed to enhanced learner motivation, based on the satisfaction of learner needs by PE teachers within the SDT framework. Similar in-service training programmes can therefore make a positive contribution to learner motivation in PE and physical activities, and indirectly to the promotion of PE and child health in South Africa.

# Abstract: O16: A 15 year historical journey of dietary/nutritional product assessment in South Africa - Consequence - Opportunity - Progress - Excellence

**Published:** 2019-01-01

**Authors:** Gary Gabriels

**Affiliations:** Department of Pharmacy and Pharmacology, Faculty of Health Sciences, University of the Witwatersrand, Johannesburg, South Africa

## Introduction

Dietary/Nutritional supplement legislation and regulation in South Africa, and elsewhere, is usually embedded in other forms of legislation rather than a standalone entity. The Constitution of South Africa sometimes obliges, parliament to legislate on certain commodities. The rapid increase in supplement sales and consumption in South Africa can be attributed to aggressive marketing by manufacturers whose claims are not always supported by published peer-reviewed evidence. The situation is further exacerbated by pressure placed on certain groups to use supplements, such as sports people, both those in their development phase to the those who are competitive, the recreational user, and extending to the elderly. As a consequence the supplement industry has grown to meet the increasing demand. These supplements may contain adulterated substances that may potentially have harmful short - and long-term health consequences. The regulation of the supplement industry is unrefined, which increases the risk of the nutritional supplements being contaminated. Contamination may be intentional, where the companies ‘spike’ their products with an ergogenic aid, or unintentional. As a result of poor regulation, contaminants or adulterants in supplements may also cause insidious effects unrelated to the listed ingredients. The aim of this paper is to present a historical trajectory and impact of the research over the defined period.

## Methods

Selected papers which formed the basis for research into dietary/nutritional supplements will be covered.

## Results

The results will focus on selected aspects of dietary/nutritional supplements, related to, consumer protection, labelling information, gymnasium users attitude, protein essences ‘melamine’, anti-depressant fluoxetine, trace element and heavy metals, and the role of health and wellness provides, linked to consequence, opportunity, progress and excellence to the overall research project.

## Conclusions

The research platform provides opportunity for interdisciplinary, transdisciplinary, multidisciplinary, research and academic collaboration and co-operation. Further, the findings over this period provide sufficient evidence for a way forward to implement a system of improved regulating, monitoring and enforcing the quality control of dietary/nutritional supplements in a more assertive way. That health and wellness providers should have or develop an explicit understanding for the potential adverse effects related to dietary/nutritional supplements, including drug-supplement interactions.

# Abstract: O17: Practical approach of sport injuries prevention – a Romanian perspective

**Published:** 2019-01-01

**Authors:** Georgescu Luminita

**Affiliations:** University of Pitesti, Faculty of Sciences, Physical Education and Informatics, Targu din Vale, Romania

## Introduction

With the continuous increase in the number of practitioners, currently there is also a significant increase in the risk of injury due to sport activities, whether they is for performance or leisure. Continuous adaptations and improvements of the equipment, of the training techniques and regulations are some of the concerns that specialists in the field of sport do have in respect with the prevention of specific injuries.

## Methods

Health status screening, medical forms and certificates to approve sports participation are some of the methods used by the sports medicine staff to prevent injuries and the risk of injury. We used a retrospective longitudinal study and analyzed the medical records of 1200 athletes (300 playing individual sports and 900 practicing sports games) over a period of five years. Through a structured interview we gathered data of the athletes’ health status and also their injury records during training and competition. We were interested also to range the athletes’ characteristics depending on their anthropometric measurements and physical development, clinical and paraclinical examinations and aerobic and anaerobic exercise capacity. Our aim was to identify the biological profile of athletes at high risk of injury and the correlation of individual sanogenic factors with the sport’s requirements, respectively the training stage. Starting from this, we wanted to project an intervention strategy, based on a combination of methods to increase the fitness level, pharmacological treatment, hygiene-dietetic measures and physiotherapy programs.

## Results

After processing all data, some injury-associated risk factors were identified: deficiencies in the athletes’ selection by sports branches; inconsistency between the physical development of the athletes and the sport requirements; paucity of the body’s ability to adapt to the physical effort according to the stage of sports training.

## Conclusions

There is a close correlation between the initial clinical and paraclinical assessment and then the periodical assessment in determining the athlete's health status and his effort adaptation ability. Moreover, the correction of biochemical imbalances has a special role in preventing the risk of injuries.

# Abstract: O18: The possible effects of biological maturation on the physical activity levels of 12–13 year-old boys and girls

**Published:** 2019-01-01

**Authors:** Barry Gerber, Anita Pienaar

**Affiliations:** 1 North-West University South Africa

## Introduction

Research on the relationship between physical activity, maturity, health and quality of life has been thoroughly researched worldwide on adults, although research on this topic has been less priority in terms of children and adolescents. Current literature indicates that boys’ and girls' physical activity decreased on average by 10% and 15% respectively as they get older. To investigate the current state of physical activity of 12–13 year old boys and girls and to determine the possible relationship between biological maturation and physical activity level of both gender

## Methodology

A convenience sample (586 participants, 303 boys and 283 girls) with a chronological age of 13.07 years from 20 selected schools in the Northwest Province, South Africa was used. Physical activities and biological maturation data was obtained through a physical questionnaire and anthropometric measurements. Descriptive statistics was used to determine means, minimum, maximum and standard deviation. Independent T-test was used to compare gender differences. One way ANOVA with a Tukey Post Hoc adjustment was carried out to determine significant differences between maturation groups and effect size was calculated by Cohans d-formula.

## Results

Boys and girls showed similar anthropometric characteristics with boys on average being taller, heavier and showing a taller sitting height. Early maturating groups on average contained taller and heavier participants in both genders accept for late maturating girls being the heavier group. Various statistical (p0.05) and small to medium practical (0.2d0.53) significant differences were found between genders physical activity levels, with boys showing higher physical activity levels throughout. On average small to medium correlation (0.1r0.57). Apart from two variables in the group of girls no other statistical significant (p>0.05) intergroup differences regarding physical activity were found.

## Conclusion

Physical activity levels at 12–13 years of age does not differ considerable between genders although boys perform better than girls. Maturity however does play an important role in boys’ and girls’ physical activity at this age especially in boys due to them entering the puberty phase.

# Abstract: O19: Creating a holistic University wellbeing strategy that connects and inspires staff and students

**Published:** 2019-01-01

**Authors:** Steve Gibling

**Affiliations:** Director Wellness and Sport Services, University of Canterbury, New Zealand

## Introduction

In 2017 the University adopted a Wellbeing Framework that amongst other things adopted an Aotearoa New Zealand focused model of interpreting wellbeing at the University of Canterbury (UC). In early 2018 the University also adopted the Wellness, Physical Activity and Sport Strategy: Te Rautaki Tākaro me te Hauora. Whilst both strategies were unique, and created a platform to strategically discuss wellbeing on campus, both strategies failed to acknowledge the role of staff in wellbeing, or their own wellbeing needs as independent but equally important players in a tertiary wellbeing approach. Both also failed to truly address the bicultural aspirations and requirements of Mana Whenua (indigenous population of New Zealand). In late 2018 a plan was established that would create a new Wellbeing Strategy which would: take account of more information related to student and staff mental health; address some of the gaps in the framework and strategy; re-engage with the student and staff population of UC and connect with allied partners in the community associated with health, wellness and wellbeing. This presentation will focus on the current wellbeing approach at the University of Canterbury, addressing why certain aspects of wellbeing strategizing have and have not worked and what UC are doing about it. In particular, the move to the use of an indigenous and holistic well-being model that will permeate every policy and practice across the University by being adopted as a part of the organisations overarching Academic Strategy. Further developments include the alignment to the Governments approach to wellbeing as a nation’s stance, with specific alignment to the United Nations Sustainable Development Goals, recognising the importance of sport and active lifestyles in well-being and post disaster response. Ultimately this is all evidenced based and a research and reporting programme is being developed that connects the internal goals right through to front line service delivery

# Abstract: O20: The effect of law changes on match loads in university rugby union players during the FNB Varsity Cup

**Published:** 2019-01-01

**Authors:** GR Gordon, H Morris-Eyton, A Kubayi

**Affiliations:** 1 Department of Human and Movement Studies, University of Johannesburg, Doornfontein Campus, South Africa; 2 Department of Sport, Rehabilitation and Dental Sciences, Faculty of Science, Tshwane University of Technology, Pretoria, South Africa

## Introduction

Rugby union is a sport which is played at a high intensity making it appealing for spectators to watch. Law changes have been implemented to make the sport more competitive, create continuity in the sport, enhance player safety and to improve the enjoyment factor for the players and spectators. FNB Varsity Cup Rugby have endeavoured to be innovative by introducing new law variations and strives to make a difference in sport. The aim of this study is to determine the effect that the law changes implemented in the FNB Varsity Cup Rugby during 2016 till 2018 had affected the players’ external load during match play.

## Methods

This study followed a longitudinal retrospective quantitative research design using secondary data from a university rugby union team. A total of 61 players’ external match load was captured on the Catapult Optimeye X4 micro-technology devices. The data was analysed and compared to each season’s data with reference to the law changes implemented during each season and match influencing factors such as match outcome, match location and quality of opponent. The tests done for the results of this research include independent t-tests, ANOVA, two-way ANOVA and Tukey HSD post-hoc analysis.

## Results

The players’ total distances, high speed running distances and PlayerLoad were more affected compared to other variables during the three seasons. The front row forwards covered the most distances in 2016 (4317±2017m) when compared to the other seasons, while the back row forwards had higher running distances in 2017 (4554±1787m) and 2018 (4426±1924m) than in 2016. With regards to the inside backs, their distances were predominantly largest in the 2017 season (5566±1852m), whereas the outside backs ran larger distances in the 2018 season (6337±737m).

## Conclusions

The back line players ran larger total distances than the forwards did during match play. Additionally, when separating the players into position specific groups, they differed in which year they ran more. It is evident that the running metrics of the players varied between each season analysed. This may indicate there is a difference between the seasons because of law variations introduced or amended.

# Abstract: O21: Social capital development through the songo.info cycling, academic support and life skills programme: programme leader and participant perspectives

**Published:** 2019-01-01

**Authors:** CW Neely, HW Grobbelaar

**Affiliations:** 1 Department of Sport Science, Faculty of Medicine and Health Sciences, Stellenbosch University, South Africa

## Introduction

The Songo.info cycling, academic support and life skills programme based in Kayamandi, Stellenbosch aims to develop confident, well-rounded, gainfully employed individuals who contribute to the community. Development through sport programmes is a popular tool to enhance social capital (the network of relationships that surround an individual), and plays an important role in marginalized societies in assisting the youth to complete school, develop transferrable life skills, enter the job market etc.

## Methods

This study explored the development of social capital through this programme from the perspectives of five programme leaders (*M* age: 40.6 years, range: 27–72 years) and nine current and past programme participants (*M* age: 18.3 years, range: 15–24 years), who took part in semi-structured individual interviews. The elicited information was analysed through qualitative content analysis utilising manual, open and negotiated coding. The authors collectively grouped the agreed-upon codes into various categories of codes, subthemes and themes.

## Results

The three themes (and subthemes) were: 1) education (world experiences, furthering education, and access to resources), 2) networks (social skills, personal relationships, and professional relationships), and 3) holistic development (physical, social and emotional domains).

## Conclusions

The network of relationships established through involvement with the programme appears to positively impact the everyday life of both the programme leaders and participants. Furthermore, the educational and holistic development seems to enhance the social mobility of the programme’s participants.

# Abstract: O22: Kinematics and muscle activation in adolescent tennis players with and without lower back pain

**Published:** 2019-01-01

**Authors:** EE Immelman, B Olivier

**Affiliations:** 1 Physiotherapy Department, Faculty of Health Sciences, University of the Witwatersrand

## Introduction

Injury in athletes can cause long periods away from active training and if not addressed correctly, may lead to recurrent injuries or chronic pain. The most powerful and strenuous stroke in tennis is the serve. Motor control is crucial during a force-generating stroke such as the tennis serve. Motor control is the result of a harmonious co-activation relationship between different muscle groups. The aim of this study was to compare the muscle activation and three-dimensional joint angles of the lower trunk and hips during the different phases of the tennis serve in adolescent tennis players with and without lower back pain.

## Methods

This study used a cross-sectional, observational design involving junior tennis players. Physical testing incorporated three-dimensional motion analysis (inertial measurement system) and wireless surface electromyography during the tennis serve. A total of six service motions were recorded and used for analysis for each participant. Kinematic analyses of the lower trunk and bilateral hips were conducted. The rectus abdominis, external obliques, erector spinae and gluteus maximus muscles were evaluated during the tennis serve. The Mann-Whitney U test was used to determine statistically significant differences between independent variables. A Pearson bivariate correlation analysis and binary logistic regression model determined which of the variables was the strongest at predicting whether a participant would be categorised into the Lower Back Pain (LBP) or No Lower Back Pain (NLBP) groups.

## Results

A total of 33 adolescent tennis players from the North Gauteng Tennis Club participated in this study. The sample comprised of 21 participants in the NLBP group and 12 participants in the LBP group. The logistic regression displayed three key points that could assist in the identification of risk factors for LBP during the tennis serve. Firstly, a smaller angle of hip flexion (start point OR=0.75; ball release point OR=0.62) and external rotation (ball release point OR=1.406; loading point OR=0.866) during the preparation phase; Secondly, a greater degree of adduction (final point OR=0.533), internal rotation (final point OR=1.252) and flexion (final point OR=1.269) of the hip during the deceleration phase; Thirdly, the importance of correct muscle activation of the anterior trunk and control throughout movement during the loading point of the preparation phase (rectus abdominis OR=0.645; external obliques OR=1.101) and final point of the deceleration phase (rectus abdominis OR=1.537).

## Conclusions

The kinematic-related key points emphasise the importance of force transfer over a stable base of support. The muscle-activity key point emphasises the importance of muscle strength and the global stability functional testing. An evaluation of the tennis serves showed that the two groups displayed different preparation and execution strategies. The identification of variables that have an effect on lower back pain status could play an important role in the prevention of injury and in rehabilitation programmes. The three key points need to be kept in mind in the treatment and training of adolescent tennis players.

# Abstract: O24: Anterior knee pain, risk factors and impact on the quality of life among runners in under-resourced communities, Ekurhuleni

**Published:** 2019-01-01

**Authors:** Siyabonga Henry Kunene, Serela Ramklass, Nomathemba Patricia Taukobong

**Affiliations:** 1 Physiotherapy Department, University of the Witwatersrand, Johannesburg, South Africa; 2 School of Clinical Medicine, University of KwaZuluNatal, Durban, South Africa; 3 Institutional Planning Department, Sefako Makgatho Health Sciences University, Pretoria, South Africa

## Introduction

Anterior knee pain (AKP) is the most problematic symptom among many runners. Due to poor rehabilitation services in under-resourced communities, runners are likely to report poorer health outcomes compared to other communities. Various factors predispose runners to AKP making a holistic rehabilitation inevitable. The purpose was to determine the presence of AKP & risk factors & its impact on the QOL among runners in in under-resourced communities, Ekurhuleni.

## Methods

This was a cross-sectional study. Convenient sampling was used to recruit participants aged between 13 & 55 with no history of knee surgery, traumatic or degenerative knee conditions. Prevalence of AKP and its risk factors were determined among 183 participants. QOL was determined among 73 participants who presented with AKP. Data collection tools included: Kujala questionnaire for AKP prevalence, developed questionnaire for extrinsic risk factors, 12 physical screening tests for intrinsic risk factors & SF-36 questionnaire for QOL. Ethical clearance, permission from 6 clubs & consent from participants were obtained. The SPSS was used to analyse data.

## Results

40% of participants presented with AKP. AKP was associated with age (X^2^=6.484, *p*=.04) & running experience (X^2^=8.389, *p* =.04). The following risk factors contributed significantly to AKP: tight hamstrings (OR=1.02; *p* =.05); tight iliotibial band (OR=1.1; *p* =.05); weak quadriceps (OR=.15; *p* =.04); weak hip muscles (OR=1.13; *p* =.00) & patellar tilt abnormalities (OR=1.33; *p* =.01), years of running experience (OR=1.72, p=.05), endurance training (OR=1.83, p =.05) & downhill running (OR=1.88, p=.04). The lowest SF-36 mean scores were found in 2 health domains: role limitation due to emotional problems (59) & vitality (59). Highest scores were found in the general physical functioning domain (72). Females presented with lowest scores (48) on role limitation due to emotional problems with noticeable difference (p=.03). Youth presented with lowest scores (62) on the social functioning domain (p=.00). Significant differences were noted on SF-36 scores between running experienced groups on the following domains: physical functioning (p=.03), role limitation due to physical problems (p=.01), vitality (p=.00), general mental health (p=.00) & social functioning (p=.00). The most affected was the group with 3–5 years of running experience which presented with scores ranging between 46 & 65. Significant mean differences were also noted between BMI groups in the social functioning domain (p=.01) where overweight & obese groups were mostly affected by AKP.

## Conclusions

A substantial presence of AKP & its risk factors were found among participants. These outcomes suggested that intrinsic & extrinsic risk factors should be considered when managing AKP among runners. This study also highlighted a need to also address non-physical features, such as the psychosocial, emotional & mental factors when formulating strategies to improve QOL among the running population. A multidimensional community-based rehabilitation programme is therefore recommended to prevent and rehabilitate AKP among runners.

# Abstract: O25: Rehabilitation approaches to anterior knee pain among runners: a scoping review

**Published:** 2019-01-01

**Authors:** Siyabonga Henry Kunene, Serela Ramklass, Nomathemba Patricia Taukobong

**Affiliations:** 1 Physiotherapy Department, University of the Witwatersrand, Johannesburg, South Africa; 2 Physiotherapy Department, University of KwaZulu-Natal, Durban, South Africa; 3 Institutional Planning Department, Sefako Makgatho Health Sciences University, Pretoria, South Africa

## Introduction

Anterior knee pain (AKP) is the most common clinical presentation among athletes accounting for 15 – 45% and it presents a threat to their quality of life. Causes and management of it remain controversial due to the lack of consensus among clinicians and researchers. The evidence for rehabilitation strategies among runners with AKP is limited and scatted. Therefore, the purpose of this study was to map the range of non-surgical and nonpharmaceutical rehabilitation approaches to AKP among runners.

## Methods

A scoping review was conducted. The five stages that were followed are: 1) defining the research question; 2) identifying relevant studies; 3) selecting a topic for the study; 4) charting and collecting the data; 5) summarising and reporting the results. Included in the study, were the original articles, written in English, included articles looked at rehabilitation strategies for AKP among runners prior to November 2017. Six electronic databases were searched, namely EBSCOHOST, CINAHL, SPORTDISCUS, PUBMED, COCHRANE, and SCOPUS.

## Results

Thirteen (13) out of 1334 articles met the inclusion criteria. Two reviewers independently participated in the screening and extraction of articles. The identified articles included 4 RCTs, 1 systematic review, 4 observational studies, 1 cohort study, 2 case studies and 1 quasi experimental study. The following rehabilitation strategies were found to be useful in rehabilitating runners with AKP: education (on symptoms and management of AKP), gait re-education (hip control, forefoot strike pattern), exercise (strengthening, stretching), foot orthoses (medially wedged insoles) and multimodal rehabilitation (combination of modalities including gait retraining, lower limb strengthening exercises, core strengthening exercises, motor control exercises and advices).

## Conclusions

This study has provided a range of rehabilitation strategies which were found useful in the prevention and rehabilitation of AKP. Anterior knee pain is a multifactorial condition that require a comprehensive but yet individualised management approach. Therefore, more comprehensive intervention studies are needed to address all the physical and non-physical needs of runners with AKP. The outcomes of this study make explicit the usefulness of the identified rehabilitation strategies among runners with AKP. These will guide clinicians in the development of rehabilitation programmes for runners.

# Abstract: O26: Education for leisure and the use of comic books in Physical Education classes

**Published:** 2019-01-01

**Authors:** Cinthia Lopes da Silva, Silvio Rossi Filho, Ana Carolina Capellini Rigoni, Luciene Ferreira da Silva, Priscilla Pinto Costa da Silva, Gisele Maria Schwartz

## Introduction

The rates of school dropouts are high in Brazil, mainly in the last years of junior high school. Most students dislike Physical Education classes because of content repetition, unsatisfactory curricular organization, among other factors. The problem gets worse when research data about the practice of physical activities by Brazilians show that a significant number of people do not get involved in any type of physical activities in their spare time (45,9%). This problem related to the access of people to physical activities in Brazil is due to matters relating to public policies of sports and leisure as well as the experiences those people had during their lives, which did not contribute in any way to the habit and/or enjoyment of activities such as sports, games, dancing and so on, which are elements that could have been practiced in their spare time throughout their lives. In addition to the biological consequences, there is a loss, which is often overlooked, and it is related to the lack of development of knowledge about the body and other cultural elements of bodily movement, which can lead people to a lack of understanding of themselves, their own sensitivity, their own emotions which can be felt and manifested through their bodies. Therefore, it is necessary to gather elements and methodological resources in order to motivate students to develop a liking for knowledge at school, and specially, for Physical Education classes, which will make them willing to participate in the proposed bodily experiences from the physical activities. It is necessary to bring to class the topic of physical activities and the construction of comic books can be a strategy to deal with presented data about the school failure and the percentage of people who do not have any kind of physical activities incorporated in their daily routines. This work has the objective of executing, describing and assessing the efficiency of a proposal for Physical Education classes, which include various contents of bodily culture of movement and the use of comic books.

## Methods

Both bibliographic and field researches were carried out and this is a qualitative study. Ten classes were given to a group of ninth graders from a public school in the city of Piracicaba/SP/Brazil.

## Results

The results show that the use of comic books as a teaching resource is powerful in that the experiences they had were felt and mediated through their “rational” and “sensitive” knowledge about physical activities and comic books. The pedagogical experiment made possible for the students of this investigation the education for leisure, the awakening to the enjoyment and knowledge about elements of the bodily culture of movement and the access to some of the leisure contents presented here, mainly by the physical, sporty and artistic, and also by the experiences in class and the construction of comic books. This work is part of a research sponsored by Fundacao de Amparo a Pesquisa do Estado de Sao Paulo (FAPESP)/Brazil, process 2017/13279–4.

# Abstract: O27: Experiences of young South African gymnasts, parents and coaches about the health benefits of sport participation

**Published:** 2019-01-01

**Authors:** WC Lucas, S Titus, M Young

**Affiliations:** 1 Department of Sport, Recreation and Exercise Science, University of the Western Cape, Cape Town, South Africa; 2 Alcohol, Tobacco and Other Drugs Research Unit, South African Medical Research Council, Cape Town, South Africa; 3 Interdisciplinary Teaching and Learning Unit, School of Public Health, University of the Western Cape, Cape Town, South Africa

## Introduction

Gymnastics, as a physical activity, presents young people with opportunities to learn physical skills, improve eye-hand coordination, develop gross and fine motor skills, and socially develop through engagement with other young people. An understanding of aerobic and anaerobic physical activities is important to enhance young people’s physical, emotional, cognitive and social development.

## Methods

This study investigated the perceptions of a purposively sampled group of young people in the Western Cape of South Africa about the health benefits of gymnastics participation, and reported findings from the perspectives of registered competitive gymnasts, gymnastics coaches, and parents of gymnasts (total n=34), with specific reference to the domains of physical, cognitive and socio-emotional development. A qualitative approach based on the Process-Person-Context-Time (PPCT) concept, adapted from the bioecological model, was used as the theoretical framework to underpin, analyze and interpret findings of the study. Four key informant interviews were conducted with four coaches, and five focus group discussions were held with gymnasts, parents of gymnasts and gymnastics coaches.

## Results

Thematic analysis of the participants’ responses indicated the following themes as health benefits and opportunities for children’s development through gymnastics participation: socio-emotional development with respect to positive attitude shifts, respect of peers and coaches, and physical development concerning musculoskeletal growth.

## Conclusions

Development of strength and flexibility were also found to be positive contributors to child development. Additionally, improvements in cognition which could positively influence children’s academic achievement and the learning of new movement skills, were also attributed to gymnastics participation. The implications of the findings for children’s holistic development was discussed.

# Abstract: O28:Physical activity and selected sociodemographic variables as predictors of nutritional knowledge amongst an adult South African sample of low socioeconomic status

**Published:** 2019-01-01

**Authors:** Smart Mabweazara, Sunday Onagbiye, Lloyd Leach, Lungiswa Tsolekile, Thandi Puoane

**Affiliations:** 1 University of the Western Cape (School of Public Health) South Africa; 2 University of the Western Cape (Department of Sport, Recreation and Exercise Science) South Africa

## Introduction

Nutritional intake contributes to human health and well-being, and plays a pivotal role in human growth and development. One factor that plays a pivotal role in the quality of an individual’s diet is their nutritional knowledge (NK). The aim of this study was to determine if physical activity (PA) and selected sociodemographic variables (gender, educational level, and employment status) predict NK amongst an adult South African sample of low socioeconomic status (SES).

## Methods

A cross-sectional study of 319 randomly sampled adults with a mean age of 57 (±10.43) years, who participated in the Prospective Urban Rural Epidemiological (PURE) study were purposively sampled for data pertaining to NK, PA, educational level and employment status using a semi-structured, researcher-generated questionnaire. Linear regression analysis was used to test predictive relationships with Alpha level set at p 0.05.

## Results

The results showed that for persons of low socioeconomic status, engaging in PA was a significant predictor of NK (p 0.05), when controlling for educational level and employment status. The model explained 4.6% of the variance on NK.

## Conclusions

In conclusion, engaging in PA was a significant predictor of NK for persons of low SES. Public health practitioners are encouraged to develop interventions that that promote PA as a means to enhancing NK. Interventions should be specifically aimed at persons of low SES who are more prone to engaging in sedentary, health-compromising behaviours. Additionally, care should be taken to contextualise the interventions for persons of low SES, so that they fit into the socioeconomic and cultural frame of reference of the participants.

# Abstract: O29: Guidelines for leadership development using leisure education for youth with disabilities in South Africa

**Published:** 2019-01-01

**Authors:** Malema Makhaya, Wegner Lisa, Young Marie

**Affiliations:** 1 Department of Sports, Recreation & Exercise Science; University of the Western Cape, South Africa; 2 Department of Occupational Therapy. University of the Western Cape, South Africa

## Introduction

Life transition occurs to everyone when they assume new roles in their lives; this may also cause a change in leisure behaviour. Life transitions can be in a form of interventions, hence, the South African government encourages a national and global intervention that would see people with disabilities being included in mainstream society activities. The latter advocates equal opportunities, living independently, having education, employment, and social integration. However, equal opportunities for people with disabilities have not been achieved due to unbalanced and inadequate resources in communities. The current study is aligned to the National Development Plan, and proposes that through leisure education, youth with disabilities will increase and sustain their leisure knowledge, make their own opportunities for leisure and become independent to their own social needs; thus developing leadership. Therefore, the aim of the study is to gain an understanding of leisure education as a tool for leadership development among youth with disabilities, in order to develop guidelines for use in the South African context.

## Methods

Using a sequential explanatory mixed methods approach, this study will consist of a systematic review, quantitative cross sectional design and a qualitative descriptive design. The study will include eight centres for physical disabilities in Cape Town, with participants aged 18–34 years. The combined population for youth with disabilities at the eight centres is 150, therefore, by applying Slovin’s formula, the sample size for this study is 109. A random and stratified sampling method will be used to recruit participants for the study. The stratifying characteristics for the sample are gender (male and female), age, and physical disability. Phase 1 of this study is a systematic review which addresses objective 1, which is to investigate how leisure programmes have been used to develop youth leadership among youth with disabilities. Phase 2 of this study, addresses objective 2: describe how youth with disabilities’ perceive leisure education and leadership development. Phase 3 of the current study addresses objective 3 which seeks to explore how leisure education can be used as a tool to develop leadership among youth with disabilities. Phase 4 will integrate finding from phase 1–3 to develop guidelines for leadership development among youth with disabilities using leisure education as a tool. Data collection methods include the Leisure Education survey developed by Sivan, a Youth Leadership Development questionnaire developed by Eikenberry, and semi-structured interviews. Data will be analysed using quantitative and qualitative methods, and the findings will be integrated to develop guidelines for leadership development through leisure education.

## Conclusions

This study will provide insight on how to empower youth with disabilities, by providing youth leadership through leisure education. Throughout, this study, participants will be exposed to, and learn, varied skills which can be applied in their daily lives into mainstream society. Through skills learnt and opportunities presented to participants in this study, there is a potential outcome for transformation among youth with disabilities. This study also adds to development and promotion of a growing capacity of youth with disabilities, developing leaders working together in solving a complex national problem.

# Abstract: O30: Perceptions of adapted physical education services through the lens of Asian parents

**Published:** 2019-01-01

**Authors:** Z McClain, D Bridges, E Bridges

**Affiliations:** 1 Department of Kinesiology, California State University, East Bay, Hayward, CA, U.S.A; 2 School of Kinesiology & Nutritional Science, California State University, Los Angeles, Los Angeles, CA, U.S.A; 3 Benjamin & Yancy Foundation, Inc., New Orleans, LA, U.S.A

## Introduction

Parents’ perceptions may be viewed through a narrow lens that could possibly be impacted by cultural context, personal beliefs, limited knowledge, and lack of awareness of educational services (Jeong, Kim, & Lee, 2015; Lai & Vadeboncoeur, 2013; Lee & Park, 2016). School personnel and parents lack the development of a partnership to determine how resources for students with a disability can be effectively utilized for programming (An & Hodge, 2013; Hamlin & Flessa, 2018; Lee, Dillon, & Stewart, 2019). This study aimed to identify Asian parents’ perceptions on adapted physical education services delivered to their children with disabilities in school environments. The study highlighted both positive and negative aspects of various experiences encountered by Asian parent participants with regards to programming and services.

## Methods

This study utilized a qualitative research design (Creswell, 2013) approved by an institutional review board and participant consent. Participants were fifteen parents with a child with a disability receiving direct services (including adapted physical education) at an educational institution and were recruited from a community-based parent support program. The investigators utilized a bilingual semi-structured questionnaire, which consisted of open-ended questions, to ensure capturing the rich data of the participants. The questionnaire was validated by experts for content validity. An interpreter was used to certify the full understanding of the parents’ written explanations, when necessary. Content validity was supported with field notes taken by the investigators during the each interview. Major themes were identified through independent coding of participant responses as well as, through field notes that were derived from the investigators.

## Results

The semi-structured questionnaire, analysis, and field note depiction led to identifying four major themes as participants consistently expressed perceptions within these broader categories. These four themes consisted of 1) the lack of understanding about parents’ cultural differences, 2) the lack of goal setting for the adapted physical education curriculum, 3) limited knowledge of parental rights, and 4) noted barriers to services experienced. The information gathered from the participants resulted in a greater understanding of their cultural differences.

## Conclusions

The study examined the importance of Asian parents’ perceptions of direct school services for their children with disabilities. It is important to understand that special education services can be perceived differently based on specific cultural beliefs. This research has provided an opportunity to better understand cultural perspectives and provided necessary recommendations to bridge the gap that may exist between an educational institution culture and Asian parent culture regarding special education services. These recommendations consist of forming collaborative partnerships in schools, providing direct support for parents to continue advocating for their children, increasing the need for school personnel to understand the Asian culture, and enhancing parental training (e.g., policies and practices of special education) through the educational system. Continued research should be conducted to broaden the scope of goals to overcome perceived barriers within any culture for children with disabilities to perhaps increase the quality of their movements and daily life activities.

# Abstract: O31: Association between physical activity levels and body fat percentage in 6–8 year-old children from a Black South African Population: BC-IT study

**Published:** 2019-01-01

**Authors:** Makama Andries Monyeki, Caroline M Sedumedi

**Affiliations:** 1 Physical activity, Sport and Recreation Research focus area, Faculty of Health Sciences, North-West University, Potchefstroom, South Africa

## Introduction

Obesity and physical inactivity are global health problems in children and adults alike, with developing countries experiencing a shift from mortality predominately driven by infectious diseases to mortality driven largely by non-communicable diseases (NCDs). The aim of the study was to determine the association between objective measured physical activity (PA) levels and body fat determined by stable isotope and Bioelectrical Impendence Analysis (BIA) in 6–8-year-old children from a black South African population.

## Methods

Participants are a total of 96 children (53 girls and 43 boys; mean age of 7.7±1.23) who are part of a larger cross-sectional study aimed at determining body composition using stable isotope technique (BC-IT). Body mass index (BMI) was calculated as weight in kilograms divided by height in meters squared. Body fat percentage was assessed using stable dilution techniques (D_2_O) and BIA (Bodystat 1500 MDD). ActiGraph accelerometer (Model GT3X-BT) determine PA for a minimum of 10 hours/day for seven consecutive days. PA data was then categories into sedentary PA (<99 counts per minute), light PA (≥ 100 counts per minute), moderate PA (≥2296 counts per minute), vigorous PA (≥ 4012 counts per minute) and moderate to vigorous PA (MVPA). Descriptive statistics and independent sample *t*-test for gender difference were performed by SPSS program. Correlation coefficients (*r*) were used to determine the relationship between physical activity and body composition.

## Results

Prevalence of overweight/obesity for the total group using D^2^O was 22% and 13% by BMI and respectively; with 12% and 11% being underweight and with girls (33%) being more overweight/obesity than the boy (7%). Girls were significantly (p=0.03) more sedentary (356.14±48.66 min/week, girls’ vs 325.41±49.38 min/week, boys) and fatter (7.26±3.29kg) compared to boys (5.44±2.43kg). Boys spent significantly (p<0.05) more time in moderate and vigorous PA (48.80±13.44 min/week) than girls (65.84±15.16±15.16). Objective PA levels (Moderate, Vigorous and MVPA) were inversely associated with fat mass as determined by stable isotope (*r*= −0.27, p=0.01, *r*=−0.27, p=0.01 and *r*= −0.27, p<0.05) and BIA (*r*= −0.27, p=0.01; *r*= −0.29, p<0.05 and *r*= −0.30, p<0.05).

## Conclusions

Girls were more overweight and sedentary compared to their boys’ counterparts. Additionally, it was evident that excessive fatness negatively affected the children participation in physical activity. Given the health implications of these results, urgent strategic interventions are recommended.

# Abstract: O32: Measuring physical activity in South African grade 2 and 3 learners: a self-report questionnaire versus pedometer testing

**Published:** 2019-01-01

**Authors:** K Nolte, GF Malan

**Affiliations:** 1 Division: Biokinetics and Sport Science, Department of Physiology, Sport, Exercise Medicine and Lifestyle Institute (SEMLI), Faculty of Health Sciences, University of Pretoria, South Africa

## Introduction

The prevalence of childhood obesity is increasing in South Africa and can be linked to decreased physical activity (PA). It is therefore important to be able to accurately measure children’s PA levels as part of a holistic strategy for maintaining healthy body weight.

## Methods

The primary aim of this study was to determine whether children in grades two and three can self-report PA by means of a questionnaire. Fifty-eight participants (28 females, 30 males) from a primary school in Gauteng were recruited for the study. The participants had to wear a pedometer (Omron HJ-720) for seven days after which they completed the Physical Activity Questionnaire for older Children (PAQ-C).

## Results

The average steps per day were 9289, with weekday steps (10 219) being more than weekend steps (6795). The mean score for the PAQ-C was 3.14 ± 0.47. There was a significant moderate correlation (r = 0.49; p < 0.01) between the overall PAQ-C score and average steps per day.

## Conclusions

Therefore, the PAQ-C can be an effective way in which to gain insight into PA levels in children but should not replace objective measures of PA. The participants in this study appear to be accumulating insufficient PA over the course of the week.

# Abstract: O33: Perceptions of physical activity participation among University students living on and off campus in the Western Cape

**Published:** 2019-01-01

**Authors:** Simamkele Nyangiwe, Tshegofatso Mgwambane, Makhaya Malema

**Affiliations:** 1 Department of Sports, Recreation and Sport Sciences, University of the Western Cape, South Africa

## Introduction

Universities are said to have a responsibility to offer a holistic education and should, therefore, include ways to prevent risky behaviours, such as sedentary living. As university students are expected to make informed decisions about their future endeavours, it is suggested that physical activity be prioritised and be part of their autonomous decisions. Physical activity plays a significant role in the mental and emotional well-being of an individual, for example, depression. Literature notes that 23% of adults and 8 % adolescents’ do not meet World Health Organizations’ global recommendations on minimum physical activity for general health promotions. It is noted with concern the increase of people living a sedentary and inactive lifestyle. Furthermore, World Health Organization suggest that as countries develop economically, simultaneously, there is a noticeable decline in physical activity due to improved technology, mode of transportation, urbanisation and cultural values. Interestingly, a multi-racial study notes that students from Asian and African countries have the lowest levels of physical activity participation, whereas, white students are the most physically active.

## Methods

Purpose of the study is to explore the perceptions of physical activity participation among students in the University of Western Cape. The study will use a qualitative ethnographic design to collect data from participants using semi-structured one-on-one interviews. Purposeful sampling method will be used to select 8 participants for the study to collect data. The study interviews will be a minimum of 30 minutes, which, will be recorded using a digital recorder and transcribed verbatim. Open coding will be applied when analysing the transcribed data through ATLAS.ti software programme. Trustworthiness of the study will be accomplished using credibility, transferability, dependability and conformability methods. The study will seek permission to collect data from the University of the Western Cape research ethics committee. Participation in this study will be purely voluntary, and participants will be advised that should they wish to withdraw from the study, this will be allowed without prejudice on the study. The study will not publish participants’ personal details, and in cases of names, pseudonym names will be used in place of participants’ names.

## Results

The current study is ongoing, however, it is noted that as lifestyle changes during the university period are sustained into adulthood, students tend to engage in risky behaviours such as alcohol and tobacco use, physical inactivity, which can lead to long term negative implications for their health and poor perceptions about their body structure. This study look to explore and identify the importance of physical activity participation among university students of the University of the Western Cape.

## Conclusions

The benefits of physical activity include improved health, life satisfaction and quality of life, social relations, community cohesion, sense of community and sense of achievement. Furthermore, individuals are recommended to engage in physical activity, from moderate or vigorous intensity exercise, 3–5 days a week to experience the benefits of physical activity.

# Abstract: O34: An investigation of the association between *vitamin-d receptor* gene bsmi polymorphism, bone health status, and muscular performance in malay young female athletes and non-athletes

**Published:** 2019-01-01

**Authors:** Foong Kiew Ooi, Abu Bakar Nor Azara, Chee Keong Chen, Ravindran Ankathil, Ahmad Aizat Abdul Aziz, Jamaayah Meor Osman

**Affiliations:** 1 Exercise and Sports Science Programme, School of Health Sciences, Universiti Sains Malaysia, Health Campus, Kelantan, Malaysia; 2 Human Genome Centre, School of Medical Sciences, Universiti Sains Malaysia, Health Campus, Kelantan, Malaysia

## Introduction

This study investigated the association between *vitamin-D receptor (VDR)* gene BsmI polymorphism, bone, muscular strength and power in Malay young female athletes and non-athletes.

## Methods

Sixty Malay females aged between 15–17 years old were involved. There were 2 groups in this study: (i) athletes group with state level athletes (n=30), and (ii) non-athletes group with non-active individuals (n=30). Participants’ knee and shoulder isokinetic muscular peak torque (strength) and average power were measured using isokinetic dynamometer. Hand grip strength test was performed. Participants’ tibial and radial quantitative ultrasound measurements of bone speed of sound were measured using bone sonometer. Whole blood was collected for DNA extraction purpose. Genotyping of *VDR* gene BsmI polymorphism was performed using Polymerase Chain Reaction-Restriction Fragment Length polymorphism (PCR-RFLP) method. Chi-square test, independent t-test and one-way ANOVA were used for statistical analysis.

## Results

In athletes, bb genotype was associated with higher bone speed of sound in the arm compared to BB genotype, while Bb genotype was associated with higher bone speed of sound in the leg compared to BB genotype. In nonathletes, BB genotype was associated with higher handgrip strength compared to Bb genotype. It was also found that BB genotype was associated with greater knee isokinetic extension and flexion peak torque per body weight (strength) compared to Bb genotype. BB and Bb genotype were associated with greater shoulder isokinetic flexion peak torque (strength) compared to bb genotype in non-athletes.

## Conclusions

The present study findings imply that *VDR* BsmI Bb and bb genotypes are associated with better bone health status in Malay young female state level athletes. *VDR* BsmI BB and Bb genotype are also associated with greater muscular strength in Malay young female non-athletes.

# Abstract: O35: Effect of a 12-week aerobic exercise programme on percentage body fat, fasting blood glucose and dyspnea in insulin resistant, obese female university employees in the Western Cape

**Published:** 2019-01-01

**Authors:** Phatlane Phindile, Susan Bassett, Sunday Onagbyiye

**Affiliations:** 1 Department of Sports, Recreation & Exercise Sciences, University of the Western Cape, South Africa

## Introduction

Obesity is recognised as a risk factor for non-communicable diseases (NCDs) which has reached epidemic proportions globally, and South Africa as one of the developing countries with significant statistical representation reported for this condition. Obesity is associated with other conditions such as type 2 diabetes, hypertension and dyslipidemia which are all part of what is called metabolic syndrome. As a strategy to reduce the levels of obesity, physical activity has been introduced in the health care professions to compliment clients who are on medications for diabetes. This study seeks to investigate the effects of a 12-week aerobic training programme on percentage body fat and fasting blood glucose levels in insulin resistant, obese females UWC employees in the Western Cape.

## Methods

The study utilised a quantitative approach using a pre-test post-test design with an intervention of an aerobic exercise programme for 12 weeks. The study included 20 females who are obese (i.e. BMI > 30 kg·m^−2^with a waist circumference >88cm) and who have fasting blood glucose levels >5.5mmol·L^−1^). Participants informed prior to the commencement of the study that participation is purely voluntary, and should they chose to withdraw from the study, they can do so at any time without any penalties or lose any benefits to which they otherwise qualify. Ethics clearance was obtained from the Biomedical Research Ethics Committee of the University of the Western Cape ethics number BM17/8/11 and participants were asked to give written consent before taking part in the study.

## Results

It was hypothesised that a 12-week aerobic exercise programme will reduce the percentage of body fat, blood glucose and dyspnea in insulin resistant obese female university employees in the Western Cape. This study adds to the current body of knowledge and literature, furthermore, this study lays down a foundation for South African stakeholders and health care practitioners to implement this programme and effectively get the country moving through PA.

## Conclusions

Supervised aerobic exercise, when executed and implemented correctly, can safely decrease fatigue levels, weight, BMI, subcutaneous and abdominal girth to their participants, however no evidence from the results suggest improvements on dyspnea.

# Abstract: O36: Perception of athletes between coaches’ behaviour and athletes’ motivation on selected collegiate table tennis athletes

**Published:** 2019-01-01

**Authors:** JIES Sibal, OYS Santelices

**Affiliations:** 1 University of the Philippines Integrated School, Diliman, Quezon City, Philippines; 2 Department of Sport Science, University of the Philippines, Diliman, Quezon City, Philippines

## Introduction

The study determined the relationship of perceived coaches' behaviour and athletes' motivation. among selected table tennis athletes of the University Athletic Association of the Philippines (UAAP).

## Methods

Selected athletes from the eight(8) UAAP schools completed the 7-point Likert Scale of Sport Motivation Scale (SMS) and 5-point Likert Scale of Leadership Scale for Sports. These were utilized to determine the motivation of athletes and perceived leadership displayed by the coach. Descriptive statistics and Pearson Chi-Square were used to determine the relationship of coaching behaviour and athletes' motivation. Mann-Whitney U and Phi-Coefficient were used to analyse the relationship of other variables (age, gender, number of playing years and year level) on perceived coaching behaviour.

## Results

The result showed that democratic coaching behaviour has significant effect on athletes’ motivation namely (To KNow-IM, To Accomplish-IM, and Identified-EM). This result indicates that a democratic coach supports the athletes' enjoyment in training and discovering new techniques increases its intrinsic motivation which will be an aid for the improvement not only for the performance of athlete but as an individual. Meanwhile, the autocratic coaching behaviour showed no significant results between athletes' motivation and perceived coaching behaviour, thus, it suggests that having a coach that is controlling, one who imposed a military style of behaviour during training or competition blocks the opportunity of an athlete to have fun in trying out specific skills and/or enjoy new discoveries of techniques, change in character, change of career opportunity, or even quit from sports. In addition, there were other factors that were considered in this study such as age, gender, number of playing years and year level. These variables showed insignificant results on relationship of perceived coaching behaviour.

## Conclusions

The problem of coaches' behaviour in relation to athletes' motivation can be minimized with numerous foreign studies regarding it. The study proves this process will take a combination of time and effort on the part of coaches and athletes. The researchers believed that further investigation may contribute to the development or enhancement for both athletes and coaches

# Abstract: O37: Preparing recreation professionals: graduate attributes expected of entry-level recreation professionals in a South African context

**Published:** 2019-01-01

**Authors:** CM. Schreck, TJ Weilbach, GM Reisma

**Affiliations:** 1 Physical Activity, Sport and Recreation, North-West University, Potchefstroom Campus, South Africa; 2 Centre for Health Professions Education, North-West University, South Africa

## Introduction

The current unemployment rate of graduate students in South Africa 7.3% an increased by more than 4% in the past 10 years. This increase can be ascribed to the economic situation of the country, but more important is the youth’s lack of work experience and absence of the skills and competencies (also known as graduate attributes) demanded by employers, reducing their suitability for employment. The focus of this study was to determine the graduate attributes required in entry-level recreation professionals in South Africa.

## Methods

A ranking-type Delphi study design was used, consisting of three iterations. Ten experts from the public, non-profit and private recreation sectors whose organisations employ entry-level recreation professionals were asked to rank the graduate attributes most required in students, and evaluate the relevance of graduate attributes for a South African context. Data were analysed using descriptive statistics and inductive coding.

## Results

“Passion for the profession”, “trainability and a willingness to learn” and “communication skills” were the top-ranked attributes. Clear differences were found in the expectations from South African graduates compared with United States graduates. “Creativity”, “ability to work with groups” and “conflict management” were added for the South African context.

## Conclusions

These results clarified what is expected of graduates entering recreation careers in SA, and it is therefore crucial that institutions preparing recreation students focus on these attributes in their programmes to enhance graduate employability. Furthermore, students need a way to put their classroom knowledge to practice, to gain self-confidence and feel prepared for their initial employment in the field. It is therefore suggested that the experiential learning students are exposed to during their degree is expanded.

# Abstract: O38: Metabolic controls and prevalence of depression symptoms: effects of sixteen weeks recreational football on male students

**Published:** 2019-01-01

**Authors:** R Tomar, VC Antony

**Affiliations:** 1 Physical Education Department, King Fahd University of Petroleum & Minerals, Dhahran, Saudi Arabia

## Introduction

There is a relation between activity and health profile and mortality from the cardiovascular diseases. This is well known fact documented that physical activity affect positively several lifestyle-related diseases and cardiovascular risk factors. One of the major health problems of this century is physical inactivity. Any exercise programme must be enjoyable so that it can be successfully incorporated into person’s lifestyle. Football is associated with high-energy expenditure.^4^ If used as tool for physical conditioning, the small-sided games of football are very helpful in eliciting heart rate up to around 90–95% of maximal heart rate. Present study investigated effects of sixteen weeks of recreational football on metabolic controls and prevalence of depression symptoms in untrained males.

## Methods

Thirty five male students were selected randomly from King Fahd university of Petroleum & Minerals (KFUPM) with mean age 18.75 yrs. Participants were randomly assigned to three groups. Group one was six a side football group (SFG, n = 12), group two was four a side football group (FFG, n = 8) and third was control group (CG, n = 15). SFG and FFG were intervention groups. Three subjects from control group didn’t report for post-test. Intervention group played recreational football for 16 weeks. Football sessions were conducted on 40x30m outdoor artificial pitch. Training sessions were organized twice a week. Each session was divided into two halves of 15 minutes with 5 minute recovery. Heart rate in intervention group was monitored during all football sessions using Polar FT7. Control group subjects followed their regular routine. One way ANOVA was employed to find difference between the groups after 16 weeks of football. Level of significance was set at .05

## Results

One-way ANOVA revealed significant difference in Very Low Density Lipoprotein (*P* = 0.005), Low Density Lipoprotein (P = 0.001), Total Cholesterol (P=.002) and Triglycerides (P= .000). Significant differences were also seen in HbA1c (P=.000) Bonferroni post hoc test revealed significantly lesser mean (5.20±0.15) in FFG than SFG (5.39±0.13, p= .000). Fasting blood sugar also reduced significantly (p=.000) Bonferroni post hoc test revealed significantly lesser mean (80.91±4.35) in SFG than CG (81.20±4.71, p= .016). FFG also had significantly lesser mean (76.87±4.88) than CG (81.20±4.71, p= .000). While no significant improvement was observed in High Density Lipoprotein (P = 0.750). Symptoms of depression were also reduced (P= .035).

## Conclusions

Findings of the present suggests that recreational football training with six and four sided teams appears to be effective in bringing change in metabolic controls in untrained males. Sixteen weeks of supervised recreation, football training was effective in reducing Fasting blood glucose. HbA1c reduced significantly in both SFG and FFG group post 16 weeks. Significant reduction was seen Total Cholesterol and Low Density Lipoprotein in both SFG and FFG groups. Our training was vigorous in nature as indicated by high average heart rate during the training sessions. Further, recreation football could not affect High Density Lipoprotein significantly. There was also reduction in depression symptoms.

# Abstract: O39: The influence of a Mindfulness-Acceptance-Commitment Intervention program on the mental toughness, self-efficacy and anxiety of male league softball players in South Africa

**Published:** 2019-01-01

**Authors:** KB Tsebe, RL Van Niekerk

**Affiliations:** 1 Department of Psychology, University of Fort Hare, Alice Campus, South Africa; 2 Department of Human Movement Science, University of Fort Hare, Alice Campus, South Africa

## Introduction

Sport psychologists and researchers have been examining the influence various psychological factors such as mental toughness, self-efficacy and anxiety on the performance of players. The aim of this study was to explore the influence of a Mindfulness-Acceptance-Commitment (MAC) Intervention program on the mental toughness, self-efficacy and anxiety of male league softball players. The prediction was that there is be a significant effect on the three constructs measured.

## Methods

A purposive sampling of 2 teams randomly assigned to an experimental (n = 9) and control group (n = 8) from 2 different provinces, representing 17 players with a mean age of 20 years was invited to participate in the study. Players in the experiment group received a 7 weeks MAC intervention program, while the control group did not. The data was obtained using the Sport Mental Toughness Questionnaire, Rosenberg Self-esteem Scale and Sport Competition Anxiety Test. The effect of the MAC program was determined with non-parametric statistical test.

## Results

The study found that the players who were involved in the MAC program maintained their appropriate levels of anxiety, self-efficacy and mental toughness throughout the competition, while the players in the control groups anxiety, self-efficacy and mental toughness was eroded during that time. The mental toughness of the experimental group, was significantly higher than that of the control group at post-test stage.

## Conclusions

The hypothesize was partially confirmed because the MAC program made a significant difference in the mental toughness of the two groups, but not the self-efficacy and anxiety of the control and experiment group.

# Abstract: O40: Shanghai amateur games: a hallmark sports event for residents

**Published:** 2019-01-01

**Authors:** Yu Weng

**Affiliations:** College of Physical Education, Soochow University, Suzhou P.R. China

## Introduction

A hallmark sports event tends to have positive influence on both the host city and its residents. Shanghai Amateur Games is a series of games that is designed for residents in Shanghai since 2017, and now it is trying to build its own “brand” and expand its influence. In this paper we discussed the features of the development process of Shanghai Amateur Games. We also raised problems for its development.

## Methods

We searched electronic databases, including CNKI and Google Scholar, using the following keywords: *hallmark event, sports event, sports management, branding*, and *CBBE model*. We also searched the website of Shanghai Amateur Games for official documents, including *Guidebook of 2019 Shanghai Amateur Games* and other files. We visited organizers of Shanghai Amateur Games who interpret the development process of this hallmark sports event. We also designed a set of questionnaire and collected participants’ feedback of the games, with which we analysed participants’ attitude towards the games.

## Results

With information from what we have gathered and the analysis of the documents we obtained, several features of the development process of Shanghai Amateur Games are as below: Residents in shanghai has began to create an impression on the “brand” of Shanghai Amateur Games. Different from famous International games held in Shanghai such as *F1 Formula* and *Shanghai ATP 1000* which are facing professional athletes, it is a hallmark sports event that encourages local residents to participate in physical activities. After three years’ effort, Shanghai amateur games is building a “brand” belonging to this city and meanwhile promote people’s participation in physical exercise. Government is playing a significant role in the development process of building a hallmark sports event. By establishing an organizing platform, Shanghai government has gathered series of amateur games that were once held spontaneously by communities or sports agents, providing them with fund and service. Assessment mechanism is a dispensable part during the whole games development process. The assessment will evaluate the influence-professionalism- and the social and economic contribution of each game. According to the assessment results, games are divided into three levels, and games in different level will get different fund and service support from the government. Also, the assessment will collect participants’ feedback, evaluate its brand value and have an influence on the game’s future development.

## Conclusions

Shanghai is now building a hallmark sports event for residents. Government and sports companies are working together to provide better service for residents and raise their awareness of physical activities. Effectiveness is oblivious but problems also exist, such as participants’ consumption awareness during watching the games is still weak; capital resources for holding a game is limited; and games attraction to residents is not enough.

# Abstract: O41: Leisure meanings of caregivers: a case study

**Published:** 2019-01-01

**Authors:** A Fouche, JT Weilbach, FG Watson

**Affiliations:** 1 Physical Activity, Sport and Recreation (PhASRec), North-West University, South Africa; 2 Quality in Nursing and Midwifery, North-West University, South Africa

## Introduction

Formal caregivers face high workloads, long working hours, staffing shortages, and emotional stress as burdens (Rose & Williams, 2015; Whal & Newmark, 2009; White, 2008). The responsibilities and heavy workload that caregivers face may lead to psychological and physical health problems like emotional exhaustion, distress and burnout, and have substantial impacts on a caregiver’s life, including his/her leisure lifestyle (Brehaut et al., 2004; Dupuis, 2000; Hung et al., 2002). In this regard, Dupuis and Smale (2000) noted the changeability and contradictory nature of leisure meanings within an institution-based caregiving context, which indicates that the caregiving role may influence the meanings that caregivers attach to leisure. Considering the possibility that leisure meanings can be formed based on individual reasoning within the context of social reality, as proposed by Watkins (2000), it is possible that formal caregivers may attach unique meanings to leisure due to their caregiving roles.

## Methods

This study made use of a qualitative approach based on an exploratory case study design. Data were collected through semi-structured one-on-one interviews and aimed to explore the leisure meanings of formal caregivers caring for persons with disabilities in the North West province. The study population included formal caregivers employed at a full-time care centre from an under-resourced area in the North West province of South Africa (N=25). From the population, data was gathered until data saturation was reached, resulting in twelve caregivers participating in the study, which is in line with recommendations by Dick (2012). The interviews were audiotaped, then transcribed verbatim. Microscopic examination was applied by going through the raw data to help with identifying words that might have different meanings to the participants or to identify different words that might have the same meaning to them (Henderson, 2006). The data were then analysed by using ATLAS.ti as a computerised tool. Information was coded and analysed into categories, themes and subthemes until certain patterns emerged.

## Results

Eight themes regarding caregivers’ meanings of leisure emerged, including (a) time they had free from responsibilities, (b) social interaction, (c) different forms of relaxation, (d) something they do out of their free will, (e) something that can take place or can be experienced anywhere or any time, (f) personal time or escape, (g) different forms of activities they like to participate in, and h) leisure as a positive experience. From the interviews, it also became apparent that these eight themes are influenced by the participants’ caregiving roles. Although not directly related to leisure meanings, an additional result that emerged was that some caregivers considered aspects of caregiving as leisure.

## Conclusions

The findings indicate that, due to their caregiving roles, caregivers attach unique meanings to leisure. As understanding leisure meanings can play a crucial role in building theory regarding leisure and may lead to improved service delivery (Schulz & Watkins, 2007:477), these findings can be used a starting point for tailoring leisure services that are in line with caregivers’ leisure meanings.

# Abstract: O42: Student feedback and effect on different training stimuli in physical education classes

**Published:** 2019-01-01

**Authors:** Pacholek M

**Affiliations:** King Fahd University of Petroleum and Minerals, Dhahran, Saudi Arabia

## Introduction

Student feedback is considered a significant source of the quality of the teaching process. provides information about their thoughts, feelings and opinions which can significantly enhance our understanding of the teaching-learning process. The main aim of this study was to investigate how students perceived different training stimuli implemented during the physical education classes and the secondary effects of these classes on their motoric and anthropometric abilities.

## Methodology

The study utilized four resistance training stimuli such as isometric, working out on machines, aerobic exercise / HIT, dynamic with equipment and aerobic training stimuli on university students. Fourteen non-physically active male students from the King Fahd University of Petroleum and Minerals (KFUPM) were randomly chosen to act as subjects (single group) for the study (n=14; age=20.5 ± 1.5 years; weight=100.5 ± 26.42 kg; height=177.8 ± 6.45 cm; BMI=31.9 ± 8.68 kg/m^2^). The training period for the study was four days per week for four weeks. In one class (50’), two different stimuli (20’-aerobic runs together with coordination and 20’-strength training) was applied. Measurement-testing and content analysis were used for obtaining data and for evaluating data a questionnaire, mathematical, statistical and logical methods were used. The Wilcoxon-signed rank test was applied as statistical tool for interpretation of results from the motoric and anthropometric tests.

## Results

Based on the results from the questionnaire it was concluded that students enjoyed and liked the aerobic stimuli the most as they had to exert less effort, but they also ranked them as the least effective. On the other hand the programs where they had to exert the most effort like the gym was ranked as the most effective but the least enjoyable. Most of the students wanted to know more about dynamic training with equipment but from the general evaluation the least effective stimulus marked was static training and the most effective aerobic exercise/HIT training. The sample showed significant improvement in all motoric tests: sit ups (19.1 ± 4.8 vs 23.1 ± 4.4), sit and reach test (18.2 ± 8.2 vs 23.4 ± 10.0cm), 5x10m shuttle run (16 ± 2 vs 14 ± 1s) and standing long jump (153.1 ± 20.7 vs 171.9 ± 23.9cm). Anthropometric tests showed no significant change. Except for visceral fat rating (9.8 ± 7.3 vs 9.5 ± 7.1), the other indicators stayed the same: BMI (31.9 ± 8.7 vs 31.8 ± 8.4 kg/m^2^), fat % (27.3 ± 9.9 vs 27.9 ± 9.4) and muscle mass (66.9 ± 10.2 vs 66.8 ± 10.0 kg).

## Conclusion

This study provides valuable information into how more training stimuli can be effectively applied to physical education classes and how KFUPM students perceived different training stimuli. From these results we can assume that students are looking for a balance between fun and hard work.
